# Presynaptic hyperexcitability reversed by positive allosteric modulation of a GABA_B_R epilepsy variant

**DOI:** 10.1093/brain/awae232

**Published:** 2024-07-19

**Authors:** Marielle Minere, Martin Mortensen, Valentina Dorovykh, Gary Warnes, Dean Nizetic, Trevor G Smart, Saad B Hannan

**Affiliations:** Department of Neuroscience, Physiology and Pharmacology, University College London, London WC1E 6BT, UK; Department of Neuroscience, Physiology and Pharmacology, University College London, London WC1E 6BT, UK; Department of Neuroscience, Physiology and Pharmacology, University College London, London WC1E 6BT, UK; Blizard Institute, Barts and The London School of Medicine and Dentistry, Queen Mary University of London, London E1 2AT, UK; Blizard Institute, Barts and The London School of Medicine and Dentistry, Queen Mary University of London, London E1 2AT, UK; Department of Neuroscience, Physiology and Pharmacology, University College London, London WC1E 6BT, UK; Department of Neuroscience, Physiology and Pharmacology, University College London, London WC1E 6BT, UK; Department of Molecular and Cellular Biology, Harvard University, Cambridge, MA 02138, USA

**Keywords:** epilepsy, neurodevelopmental disorder, inhibition, GABA, GABA_B_ receptor, positive allosteric modulator

## Abstract

GABA_B_Rs are key membrane proteins that continually adapt the excitability of the nervous system. These G-protein coupled receptors are activated by the brain's premier inhibitory neurotransmitter GABA. They are obligate heterodimers composed of GABA-binding GABA_B_R1 and G-protein-coupling GABA_B_R2 subunits. Recently, three variants (G693W, S695I, I705N) have been identified in the gene (*GABBR2*) encoding for GABA_B_R2. Individuals that harbour any of these variants exhibit severe developmental epileptic encephalopathy and intellectual disability, but the underlying pathogenesis that is triggered in neurons remains unresolved.

Using a range of confocal imaging, flow cytometry, structural modelling, biochemistry, live cell Ca^2+^ imaging of presynaptic terminals, whole-cell electrophysiology of human embryonic kidney (HEK)-293 T cells and neurons and two-electrode voltage clamping of *Xenopus* oocytes, we have probed the biophysical and molecular trafficking and functional profiles of G693W, S695I and I705N variants.

We report that all three point mutations impair neuronal cell surface expression of GABA_B_Rs, reducing signalling efficacy. However, a negative effect evident for one variant perturbed neurotransmission by elevating presynaptic Ca^2+^ signalling. This is reversed by enhancing GABA_B_R signalling via positive allosteric modulation.

Our results highlight the importance of studying neuronal receptors expressed in nervous system tissue and provide new mechanistic insights into how GABA_B_R variants can initiate neurodevelopmental disease whilst highlighting the translational suitability and therapeutic potential of allosteric modulation for correcting these deficits.

## Teaser

Variants in GABA_B_ receptors can cause severe epileptic encephalopathy and intellectual disability. Minere *et al.* provide new insights into the mechanisms by which variants give rise to neurodevelopmental disease, and show that positive allosteric modulators have potential as a new therapy.

## Introduction

Excitability, an omnipresent feature of neurons, endows a nervous system with the ability to perform complex computational tasks. Precise fine-tuning and dynamic control prevents this ubiquitous defining feature of the nervous system from transforming into a pathological state exemplified by neurological diseases such as epilepsy. Consequently, measured activation of γ-aminobutyric acid (GABA) type-B receptors (GABA_B_Rs) by the brain's main inhibitory neurotransmitter GABA has evolved as one crucial delimiter of cellular excitation. GABA_B_Rs are class C G-protein coupled receptors (GCPRs)^1^ that inhibit adenylyl cyclase and Ca^2+^ channels and activate inwardly-rectifying K^+^ channels.^2^ Overall, these actions reduce neuronal excitability by inhibiting presynaptic neurotransmitter release and dendritic Ca^2+^ signalling in addition to increasing postsynaptic membrane conductance.^1,3^ Unsurprisingly, disruption of GABA_B_R signalling is involved in multiple neurological conditions, including spasticity, epilepsy, schizophrenia, addiction and substance abuse.^4-8^

**Figure 1:**
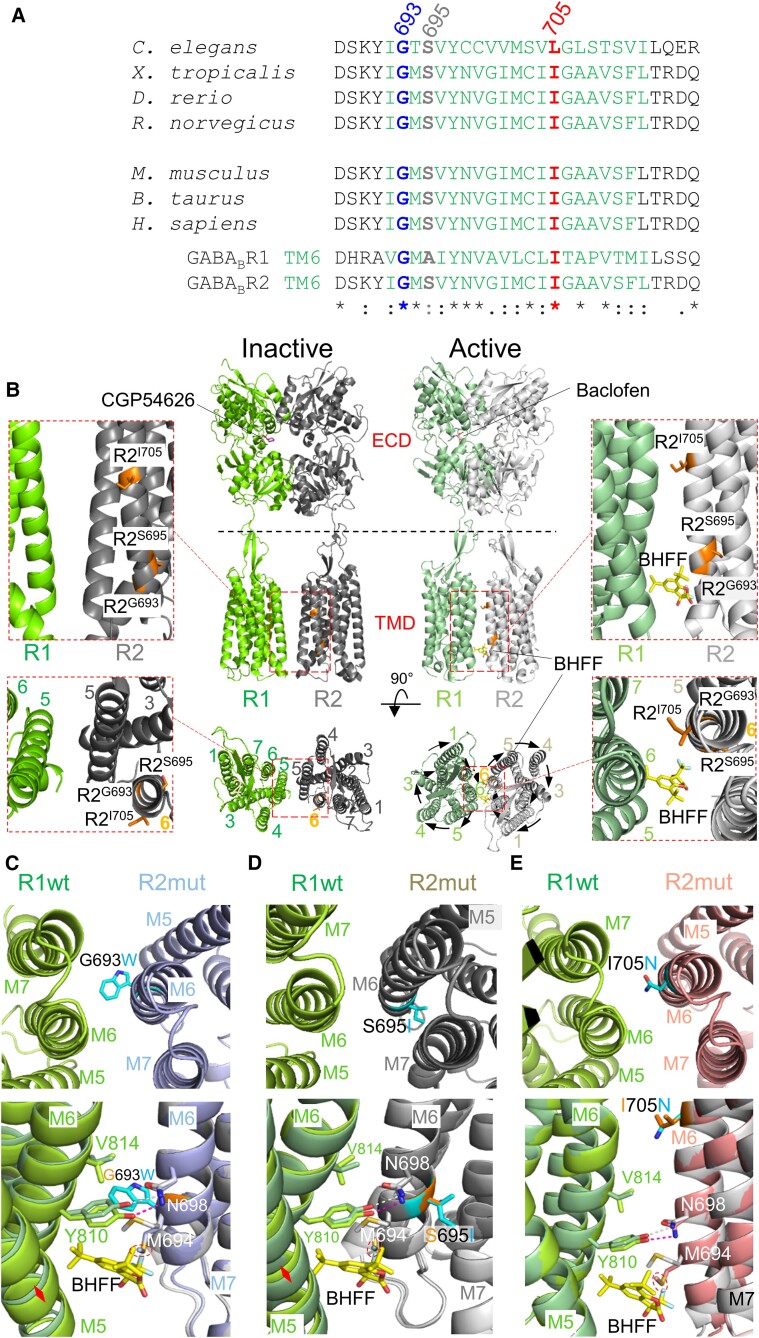
**GABA_B_R2 variants align at the heterodimer interface of the activated receptor**. (**A**) Primary amino acid sequences of GABA_B_R2 showing complete (asterisk) or high (colon) conservation of G693, S695 and I705 between species and with the sequences for murine GABA_B_R1 and R2. (**B**) Cryo-electron microscopy structures of inactive (PDB:7C7S) and active (7C7Q) GABA_B_R heterodimers, depicting transmembrane domain (TMD) α-helix 6 (M6) with locations for G693W, S695I and I705N (orange; wild-type residues depicted). Note that in the inactive structure, GABA_B_R2 M6 is positioned outside the heterodimer interface (*left*, *inset*), but due to clockwise rotations of TMDs during activation (black arrows in the active structure), M6 from R1 and R2 form the interface of the active heterodimer (*right*, *inset*). The GABA_B_R positive allosteric modulator (PAM) BHFF binds to the inter-subunit interface close to G693 and S695. (**C–E**) Structural models for G693W (**C**; cyan side-chains), S695I (**D**) and I705N (**E**), based on the active structure, 7C7Q. TMD interface representations (*top*), highlighting the side-chain position of the three mutations (cyan, with elements N: blue; O: red). The mutation models aligned to wild-type 7C7Q structure (*bottom*) with selected residues of interest and BHFF (yellow) shown. The models predict varying degrees of side-chain and α-helical shifting (red arrows) due to the mutations. H-bonds are shown as stippled lines (wild-type: grey; epilepsy mutants: pink). Predicted clashes between mutant R2 M6 94 (with sulfur atom shown in yellow) and BHFF are shown as red stippled rings.

Cell surface GABA_B_Rs are obligate heterodimers composed of two subunits: GABA_B_R1 and GABA_B_R2. Oligomerization is indispensable for function as GABA_B_R1 contains the GABA binding site, whereas G-protein coupling occurs at GABA_B_R2.^9,10^ In addition, GABA_B_R2 is necessary for cell surface expression of GABA_B_R1^11^ and ensuring cell surface stability of the heterodimer.^12^ Single nucleotide variants of the gene encoding for GABA_B_R2 (*GABBR2*) are now implicated in a wide range of neurodevelopmental disorders^13^ often sharing common symptoms, including: intellectual disability with developmental and epileptic encephalopathy and infantile seizures (e.g. T394M,^14^ G440R,^15^ M668L,^16^ G693W,^13,17^ I705N,^18^ S695I^18^); autism spectrum disorder (R212Q)^19^; and atypical Rett syndrome (A567T, A707T).^14,20-24^ Three of these *de novo* variants (G693W, S695I, I705N) that are located in the highly conserved sixth transmembrane (TM6) α-helical domain (Fig. 1A), which is critical for GPCR activation, precipitate a range of neurodevelopmental defects prominently characterized by seizures at an early age (1.5–11 months). All three individuals with these variants exhibit severe intellectual disability with no speech skills and the G693W- and S695I-expressing individuals exhibit poor posture (inability to sit up), while the carrier of I705N can only walk with support. Two of these variants (S695I and I705N) have previously been characterized in non-neuronal expression systems. Reduced signalling efficacy was observed in the absence of changes to cell surface expression.^20,22^ Currently, the signalling properties of all three TM6 variants expressed in neurons are unknown.

Given the importance of GABA_B_R2 for signalling and cell surface trafficking of GABA_B_Rs, which ultimately determines the macroscopic efficacy of GABAergic inhibition via these receptors, we used a range of approaches to characterize these developmental epileptic encephalopathy variants. We report that severe impairment of neuronal plasma membrane expression and presynaptic effects on Ca^2+^ signalling gives rise to signalling deficits that may underlie seizures and adverse neurological phenotypes. Moreover, we demonstrate that a GABA_B_R positive allosteric modulator (PAM) rescues the synaptic deficits, which has important therapeutic implications for treating individuals expressing these variants.

## Materials and methods

### cDNA, cell culture and transfection

pEGFP-C1, rat α-bungarotoxin (α-BgTx) binding site-tagged GABA_B_R1, myc-tagged GABA_B_R1a and flag-tagged GABA_B_R2 in pRK5 and synaptophysin-GCaMP6*f* have been described.^12,25^ Single point mutations equivalent to human G693W, S695I and I705N (numbering includes the signal peptide) were created for the rat—G692W, S694I and I704N—in flag-tagged GABA_B_R2 using an inverse PCR method^12^ and DNA sequencing to validate the sequences.

All work on animals was performed in accordance with the Animals (Scientific Procedures) Act, 1986. HEK-293 T cells with or without a stable transformation with Kir3.1/ 3.2 channels (GIRK cells) and hippocampal cultures prepared from embryonic Day (E) 18 Sprague-Dawley rats were grown and transfected as described.^12,25-27^

### Co-immunoprecipitation

HEK-293 T cells were lysed 24 h after transfection and incubated overnight at 4°C with Dynabeads protein G (Invitrogen; 15 μl 50% slurry) conjugated to a rabbit anti-myc antibody (Abcam, ab9106; 2.5 μl) followed by washes with a buffer containing a decreasing molarity of NaCl and processed for sodium dodecyl sulfate-polyacrylamide gel electrophoresis (SDS-PAGE) and western blotting using a mouse anti-FLAG-tag antibody (1:1000; Sigma, F1804) and horseradish peroxidase (HRP)-conjugated goat-anti-mouse antibody (1:10 000; ThermoFisher Scientific, 31430). Blots were developed using Immobilon® Crescendo western blot HRP substrate (Millipore, WBLUR0500) and imaged using an ImageQuant LAS4000 mini (GE Life Sciences) followed by band intensity analysis in ImageJ (ver 1.52p). The membranes were buffer-stripped and reprobed with a mouse anti-GABA_B_R1 antibody (1:1000; Neuromab, N93A/49) and the same HRP-conjugated goat-anti-mouse antibody.

### Whole-cell patch clamp electrophysiology

GABA- or baclofen-activated K^+^-currents were recorded 36–48 h or 5–7 days after transfection of GIRK cells and hippocampal neurons, respectively, in a Krebs saline solution and KCl-based internal solution at −70 mV holding potential as described previously.^26^ GABA_B_R PAM potentiation curves were constructed using the same approach for untransfected neurons at 14–21 days *in vitro* (DIV) or GIRK cells 36–48 h after transfection.

Neuronal GIRK current recordings were performed in 2 mM kynurenic acid and 25 µM picrotoxin. Baclofen current densities were calculated by dividing whole-cell K^+^ currents by cell membrane capacitance (measured by applying brief −10 mV pulses).

Concentration-response curves were generated by measuring the current (*I*) and normalizing to the maximal response (*I_max_*). *I_min_* defines any pedestal current. Data were fitted with a modified Hill equation:


$$I=I_{min}+(I_{max}-I_{min})(1/(1+{\left(EC_{50}/[A]\right)}^{n}))$$

where A is the concentration of the agonist, EC_50_ is the concentration of agonist causing 50% of the maximum response and *n* is the Hill slope.

Miniature excitatory postsynaptic currents (mEPSCs) were recorded at −70 mV using either a Cs methanesulfonate-^28^ or K gluconate-based^29^ internal solution and analysed using WinEDR (ver 4.0.2) and WinWCP (ver 5.7.0) and T_50_, rise time and charge transfer were measured from uncontaminated mEPSCs.

Action potentials were recorded at resting membrane potentials using the K-gluconate internal solution. The resting membrane potential of each cell was noted immediately after establishing whole-cell configuration in the absence of any current injection. Action potential firing rates were determined by analysing epochs of 1–2 min and kinetic properties of action potentials were measured from individual uncontaminated spikes.

### Two-electrode voltage clamp

*Xenopus laevis* oocytes were prepared for injection following removal from ovaries and dissociated by collagenase treatment as described previously.^30^ Oocytes were injected with cRNAs for GABA_B_R1 plus either wild-type or variant S695I GABA_B_R2 in equimolar ratios. Two-electrode voltage-clamp recordings were performed 3–5 days after injection at room temperature at −60 mV in a recording solution containing (in mM): 40 KCl, 52 NaCl, 5 HEPES, 1.8 CaCl_2_, 1 MgCl_2_, pH adjusted to 7.4. An Axoclamp 2B amplifier, Digidata 1322A interface, and pClamp 8 (Molecular Devices) were used for recording membrane currents.

### Immunolabelling and confocal imaging

Neurons were processed for confocal imaging at 12–14 DIV by fixation in 4% v/v paraformaldehyde for 10 min at room temperature, followed by incubation in a mouse anti-flag antibody (F1804, Sigma) and a goat anti-mouse Alexa Fluor 555 (AF555) secondary antibody (A28180, ThermoFisher), before mounting in Prolong gold (Life Tech). Confocal images were acquired using a Zeiss LSM 510 confocal microscope as described previously.^27^ Images were analysed using ImageJ (1.52p).

### Ca^2+^ imaging and analysis

Synaptophysin-GCaMP6f Ca^2+^ transients were imaged as described previously.^25^ Presynaptic terminals were identified and delineated by regions of interest (ROIs) using ImageJ (ver 1.52p) custom plugins and the particle analysis function. Fluorescence intensity within individual puncta was measured and the fluorescence signal (F) normalized to baseline fluorescence (F_0_) to obtain ΔF/F_0_. Ca^2+^ transients less than 3× the signal-to-noise ratio were excluded from the analysis. The ΔF/F_0_ values were exported as text files using custom Visual basic plugins and imported into WinEDR (ver 4.0.2), where ΔF/F_0_ peaks were detected and processed for analysis using WinWCP (ver 5.7.0).

### Structural plasticity of dendritic spines and morphology

Enhanced GFP-expressing live hippocampal neurons were imaged at 12–16 DIV in Krebs. The main apical dendritic segment was imaged in 3D for neurons with stereotypical pyramidal morphology, whereas the thickest dendrite was selected for analysis in neurons displaying non-pyramidal morphology. Dendrites were imaged in optimized *z*-thickness using a Zeiss LSM 510 microscope and a 40× water objective. Dendritic spines were analysed using Neuronstudio (ver 0.9.92).^31^

### Flow cytometry

Expression levels of GABA_B_R subunits were analysed 48 h after transfection using a Becton Dickinson Aria IIIu flow cytometer for live or 0.1% Triton-X permeabilized HEK-293 T cells as described^30^ using a mouse anti-flag antibody (F1804, Sigma) and a goat anti-mouse Alexa Fluor 647 (AF647) secondary antibody (A21235, ThermoFisher) or α-BgTx coupled to AF555 (Thermofisher, B35451).

### Homology modelling

We compared cryo-electron microscopy (cryo-EM) structures of GABA_B_Rs in: a presumed apo-state, PDB 6VJM^32^; several inactive states, 6WIV^33^; CGP54626-bound, 7C7S^34^ and CGP55845-bound states, 6W2X^35^; baclofen and rac-BHFF activated state, 7C7Q^34^; and an SKF97541 and GS39783 bound active state, 6UO8.^32^ These state-dependent structures revealed high levels of structural similarity and we selected two structures exhibiting the highest spatial resolution, 7C7S: 2.9 Å and 7C7Q: 3.0 Å,^34^ to represent an inactive and active state of the GABA_B_R, respectively. Wild-type residues were substituted for variants using PyMol (ver 2.5.4) and receptor models generated using Modeller (v10.4).^36^ We generated 50 models for each mutation based on 7C7Q (activated) to explore how the amino acid substitutions may disrupt receptor structure and thus the function of the activated state. The models were ranked by energy analysis using QMEANBrane^37^ to identify the most optimal model. This model was analysed in Scwrl4^38^ to optimize the positioning of side-chain rotamers, before processing in MolProbity^39^ for all-atom structure validation, where erroneous Asn, Gln and His rotamers were corrected. Next, the structure underwent minimization in Chimera 1.16^40^ before a final run in MolProbity to ensure that each optimization step had indeed improved the structural integrity of the receptor models.

The effect of the variants on GABA_B_R2 transmembrane domain (TMD) structure was assessed by aligning both mutant and wild-type structures. To investigate whether G693, S695, I705 (wild-type R2 structure; 7C7S) or G693W, S695I, I705N (variant R2 structure based on 7C7Q) may engage in H-bond formation, cation-π or π-π interactions, all neighbouring residues to the variants within a radius of 50 Å were analysed.

### Statistical analysis

Data compliance with a normal distribution was tested using GraphPad Instat (GraphPad). For such distributions, a two-tailed unpaired *t*-test or a one-way ANOVA (Tukey–Kramer test) was used to compare 2 or >2 samples, respectively. Repeated measures ANOVA (Tukey–Kramer test) was used to compare the normally distributed conditions applied to the same cell. For non-normally distributed data, a Mann–Whitney rank-sum test or Kruskal–Wallis (KW) one-way ANOVA (with Dunn’s test) was used for 2 or >2 samples, respectively. Data in bar charts represent mean ± standard error of mean and box plots show median, 25%–75% interquartile range and 5%–95% whiskers.

## Results

### Variants cause structural changes to R2 subunit transmembrane domains

Class-C GPCR TMDs transduce agonist binding signals from the orthosteric extracellular domain (ECD) binding site to G-protein-activating intracellular loops.^1^ Since TMD amino acids can profoundly affect GPCR signalling,^41,42^ the locations of Gly693, Ser695 and Ile705 within the TM6 of GABA_B_R2 were studied using homology modelling. Comparing cryo-EM heterodimer structures for inactive (PDB: 6VJM,^32^ 6WIV,^33^ 7C7S^34^, 6W2X^35^) and active states (7C7Q^34^, 6UO8^32^) confirmed the TM6 localization of these variants. Moreover, upon activation, the TMDs rotate clockwise, shifting the heterodimer interface from predominantly TM5 to TM6,^43^ thus bringing both GABA_B_R1 and GABA_B_R2 TM6 α-helices into close proximity (Fig. 1B).

From this apposition, G693W introduces a bulky tryptophan directly into the dimer interface physically disrupting the TMD α-helical structure (7C7Q; Fig. 1C). Furthermore, for S695I, the non-polar isoleucine is orientated away from the heterodimer interface projecting laterally towards GABA_B_R2 TM7 shifting the relative positions of the α-helices (Fig. 1D). We predict that such lateral shifts are likely to affect functional coupling of ligand-binding to G protein activation due to suboptimal α-helical positioning ultimately disrupting GABA_B_R physiology. For I705N, as noted for G693W, the mutant residue also inserts directly into the inter-subunit space, but here the TMDs are physically unaffected due to the smaller side-chain volume (by 36%) between Ile and Asn; however, hydrophobic Ile is replaced by Asn with its polar amino group (Fig. 1E). Subsequent side-chain rotamer optimization revealed considerable orientation differences in the TMD interface between R2 wild-type and variants. By using computational model predictions for the relative positioning of the three R2 variants, we observed that GABA_B_R2 M6 94, which co-ordinates the binding of PAMs rac-BHFF and GS39783,^34,44,45^ is now repositioned into the PAM binding cavity. This feature of M694 is apparent with all three R2 mutants. We predict that this will result in residue-PAM molecule clashes and therefore suboptimal PAM binding (Fig. 1C–E).

Together, these results suggest that the mutations introduce a number of structural changes in GABA_B_Rs focused on the critical TMD. Notably, prominent changes to the TMDs occur with G693W and S695I, and side-chain orientations are reconfigured in all three variants, principally affecting the TM6 subunit interface.

### Impaired GABA-activated signalling of GABA_B_R variants

**Figure 2:**
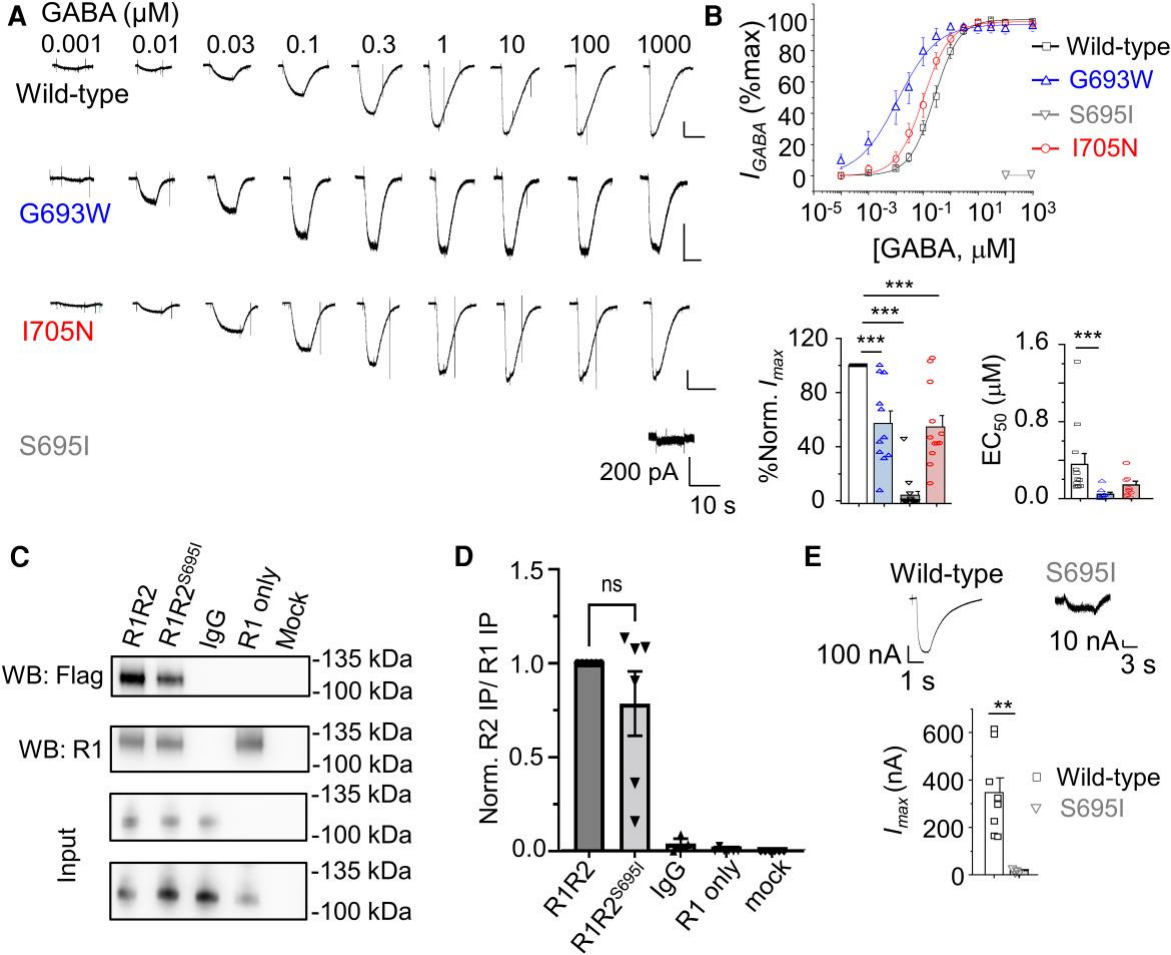
**Reduced maximal currents of GABA_B_R variants expressed in GIRK cells.** (**A**) GABA-activated currents (I_GABA_) of wild-type and variant GABA_B_R2 in GIRK cells expressed with GABA_B_R1. Note the negligible current amplitude for S695I. (**B**) Concentration response relationships, normalized (norm.) to the maximal (max) GABA currents (=100%) and EC_50_s of wild-type and variant receptors. Normalized maximal currents (%)—wild-type: 100 (*n* = 12); G693W: 57 ± 9.3 (*n* = 11); 695I: 4 ± 2.7 (*n* = 17); I705N: 54.5 ± 8.5 (*n* = 12). EC_50_s (µM)—wild-type: 0.36 ± 0.1 (*n* = 12); G693W: 0.04 ± 0.02 (*n* = 8); I705N: 0.14 ± 0.04 (*n* = 11). (**C**) Western blot (WB) of immunoprecipitated *myc*-GABA_B_R1 from human embryonic kidney (HEK)-293 cells transiently expressing GABA_B_R1^myc^ and wild-type or mutant GABA_B_R2^flag^. Immunoprecipitated samples (*top*) and input—10% of the cell lysate with corresponding expressing receptors (*bottom*)—were first probed for FLAG-tag with 1:1000 mouse anti-FLAG-tag antibody; the same membranes were stripped and reprobed with anti-GABA_B_R1 antibody. Numbers on the *right* of each blot = molecular weights (kDa). (**D**) Bar chart represents normalized band intensity of GABA_B_R2 (R2) to GABA_B_R1 (R1): R1/R2 1.00 ± 0.00 (*n* = 6), R1/R2^S695I^ 0.79 ± 0.17 (*n* = 6), R1/R2 IgG control 0.04 ± 0.02 (*n* = 4), R1 only 0.01 ± 0.004 (*n* = 6), mock transfected 0.00 ± 0.00 (*n* = 6). ns = not significant, one-way ANOVA with Tukey–Kramer test. (**E**) Example two-electrode voltage clamp recordings for 1 mM (wild-type) and 10 mM (S695I) GABA-activated currents for R1R2 wild-type and R1R2^S695I^ expressing oocytes. The bar chart shows mean ± standard error of the mean maximum GABA-activated currents (*n* = 6–8). **P < 0.001, one-way ANOVA with Tukey–Kramer test.

The pharmacological profiles of all three GABA_B_R variants were probed in GIRK cells using whole-cell electrophysiology. Sequentially expressing each GABA_B_R2 variant with GABA_B_R1 resulted in heteromeric cell surface expression for G693W and I705N, evident from functional GABA-activated GIRK currents (called GABA currents hereafter) but not for S695I, examined with up to 1 mM GABA (Fig. 2A and B). Co-immunoprecipitation of GABA_B_Rs eliminated deficits in heterodimerization as a cause for GABA insensitivity of S695I, since similar amounts of wild-type and S695I GABA_B_R2 were co-immunoprecipated with GABA_B_R1 [*P* > 0.05; *F*(4,23) = 35.5, *P* < 0.0001; one-way ANOVA; Fig. 2C and D].

Analysis of the maximal GABA-activated K^+^ currents revealed reduced maxima for both G693W and I705N GABA_B_Rs compared to R1R2 wild-type [by ∼50%; *F*(3,48) = 50.7, *P* < 0.001, one-way ANOVA] and S695I as noted, yielded negligible current (Fig. 2A and B). Interestingly, GABA potency at G693W and I705N receptors was increased (or trending) compared to wild-type receptors [*F*(2,28) = 4.1, *P* = 0.0276, one-way ANOVA; Fig. 2A and B].

Consistent with the low current levels for S695I in HEK cells, expression of this mutant in *Xenopus* oocytes with wild-type R1, and using a two-electrode voltage clamp,^30^ also revealed reduced maximal GABA current (Fig. 2E; *P* = 0.0018, two-tailed unpaired *t*-test). As a consequence, we were unable to construct full concentration curves due to the small-sized currents, although similar reduced efficacy and increased GABA potency for S695I have been reported.^46^

Collectively, these results suggest that, when expressed on the plasma membrane of cell lines or oocytes, G693W and I705N displayed impaired functional properties, whereas S695I exhibited severe functional impairment.

### Cell-type dependent plasma membrane expression deficits of R2 variants

**Figure 3:**
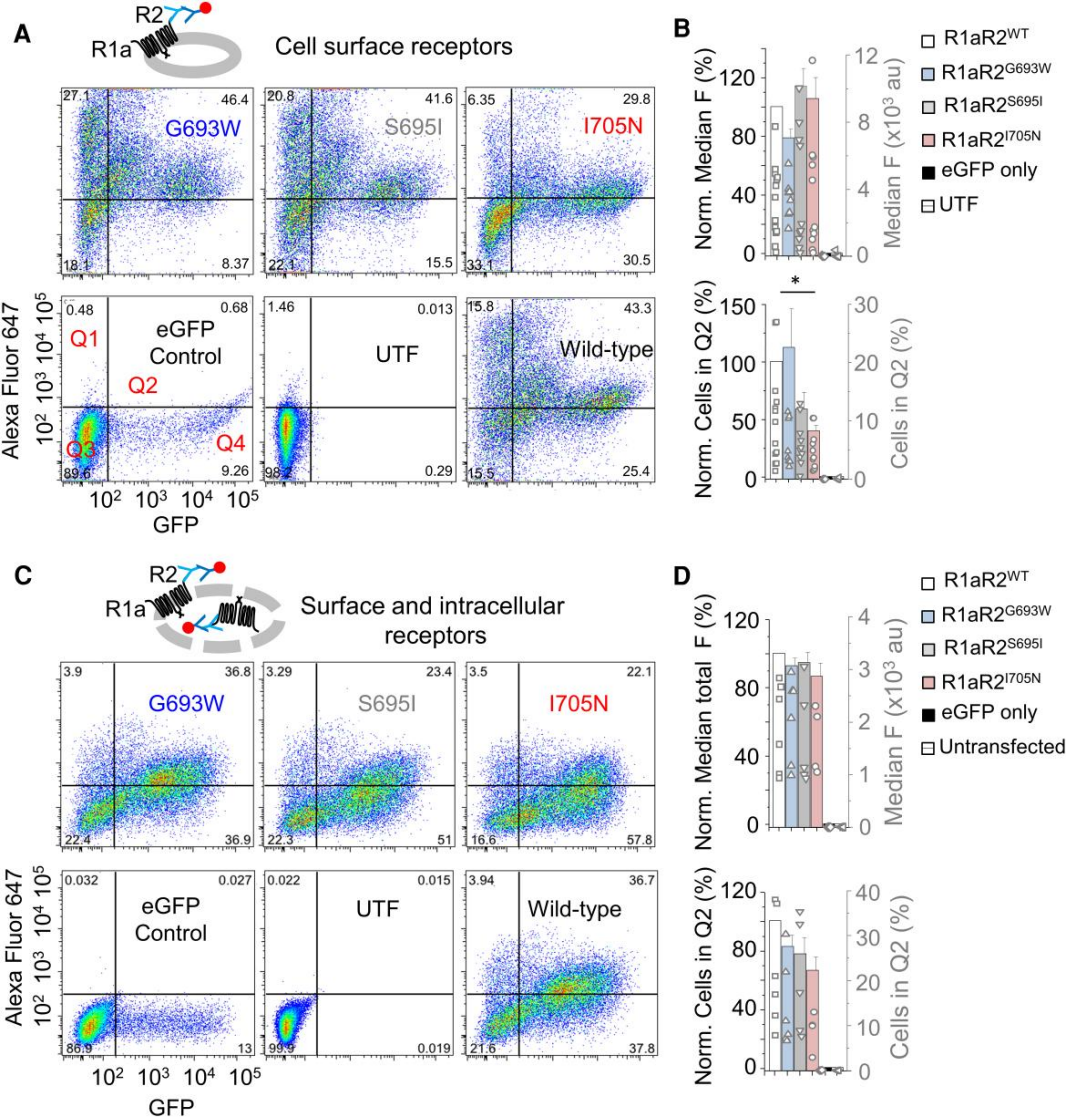
**Impaired cell surface expression for R2 variants in human embryonic kidney (HEK-293) cells.** (**A**) Cytofluorograms of cell surface staining for GABA_B_R1a with wild-type and variant GABA_B_R2 (ordinate) in GFP (abscissa) expressing HEK-293 cells. Cells expressing GFP-only and untransfected cells are also shown. Flag-tagged GABA_B_R2 was labelled with an anti-flag antibody followed by an Alexa Fluor 647 secondary antibody (pictogram). Numbers in the corners of the fluorograms are the percentage of cells in each quadrant. (**B**) Normalized (to R1R2 wild-type = 100%; left ordinate) and raw (right ordinate) cell surface fluorescence for the cells shown in **A** (*top*). *Bottom*: Percentage of fluorescing cells [normalized (left ordinate) to R1R2 = 100%, and percentage of total cells (right ordinate)] in Quadrant 2 (Q2) for the cells shown in **A**. (**C**) Cytofluorograms of total (cell surface + intracellular) R1a with wild-type and mutant GABA_B_R2 staining in HEK-293 cells. GABA_B_R2 was labelled after permeabilization (pictogram). (**D**) As for the bar graphs shown in **B**, normalized and raw fluorescence and the number of cells (%) in Q2 for wild-type, variant GABA_B_R2 in permeabilized cells, and just GFP-expressing or untransfected permeabilized cells are shown. *P < 0.05, Kruskal–Wallis one-way ANOVA with Dunn’s test.

A reduction in the maximal GABA current for GABA_B_R variants could arise from lowered cell surface receptor expression. This aspect was examined using a flow-cytometry-based immunolabelling approach^30^ measuring plasma membrane levels of flag-tagged GABA_B_R2 (GABA_B_R2^flag^) in live HEK-293 T cells co-expressing R1a subunits. The median cell surface fluorescence for variant GABA_B_R2 remained unchanged [*P* > 0.05; *F*(5,50) = 30.8, *P* < 0.001 KW one-way ANOVA; Fig. 3A and B]. However, the percentage of cells captured in the Q2 quadrant, which is indicative of cell surface expression efficiency^30^ (reflecting eGFP expressing cells that are also positive for GABA_B_R2 cell surface expression), was reduced for I705N [*P* < 0.05; *F*(5,50) = 33.7, *P* < 0.001, KW one-way ANOVA; Fig. 3A and B], trending to a reduction for S695I relative to wild-type receptors, whilst G693W was similar to wild-type (Fig. 3A and B).

These results were not due to changes in total protein expression levels (intracellular + cell surface) as fluorescence intensity [*F*(5,27) = 25.5, *P* < 0.001, KW one-way ANOVA] and the percentage of Q2 area [*F*(5,26) = 25.6, *P* = 0.0001, KW one-way ANOVA] of receptor-expressing-cells labelled following fixation, and permeabilization remained unchanged between the R2 variants and wild-type GABA_B_Rs (*P* > 0.05; Fig. 3C and D).

GABA_B_Rs are usually expressed on the cell surface as obligate R1aR2 heteromers. However, GABA_B_R2 can be expressed alone on the HEK cell surface as a homomer.^12,47^ Therefore, to assess R1aR2 expression, we quantified the expression of GABA_B_R1 in the presence of GABA_B_R2, which allows heterodimer expression to be unequivocally studied. HEK-293 T cells transiently transfected with GABA_B_R1a^BBS^ (containing an α-BgTx binding site^12^) and GABA_B_R2 were labelled with α-BgTx coupled to AF555 (BgTx-AF555). Consistent with the GABA_B_R2^flag^ labelling, cell surface expression levels of GABA_B_R1 remained unchanged (*P* > 0.05) when expressed with wild-type or variant GABA_B_R2 [*F*(5,62) = 54.2, *P* < 0.001, KW one-way ANOVA; Supplementary Fig. 1]. Remarkably, similar to GABA_B_R2, GABA_B_R1% Q2 area was lower (*P* < 0.05) when expressed with R2 I705N but unchanged for G693W or S695I (*P* > 0.05) in comparison to wild-type receptors [*F*(5,62) = 54, *P* < 0.001, KW one-way ANOVA]. Here too, in permeabilized cells, expression of GABA_B_R2 variants did not alter (*P* > 0.05) the total expression levels of GABA_B_R1 [fluorescence *F*(5,38) = 32.3, %Q2 *F*(5,38) = 32.7, *P* < 0.001, KW one-way ANOVA].

Overall, these results suggest that G693W and S695I express on the HEK-293 T-cell plasma membrane at similar levels compared to wild-type receptors, while I705N expression is reduced. Furthermore, the absence of K^+^ currents for S695I in HEK-293 cells (Fig. 2A) is not due to reduced cell surface expression and instead must arise from signalling defects caused by this variant.

**Figure 4:**
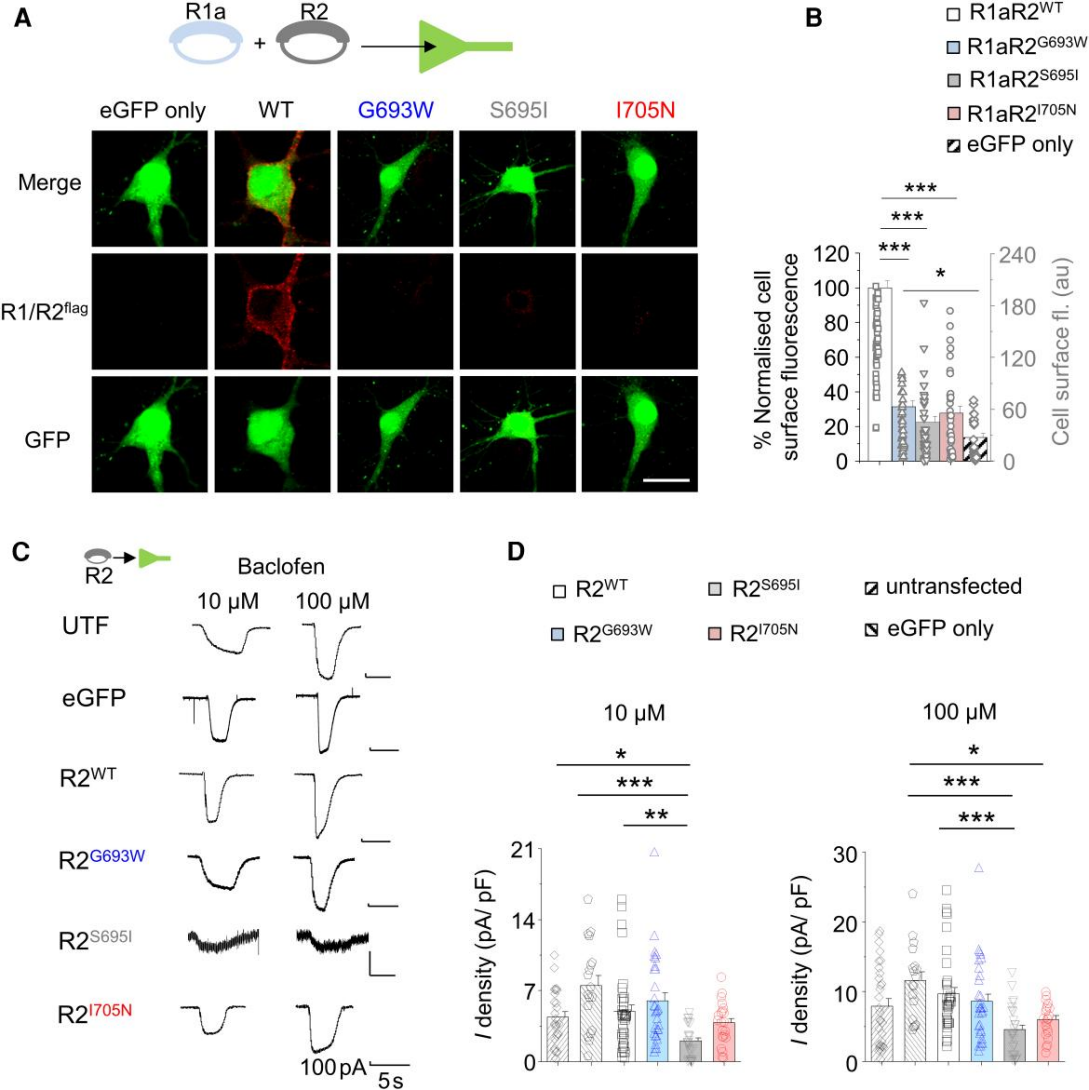
**Impaired cell surface expression and signalling for mutant GABA_B_Rs in hippocampal neurons.** (**A**) Confocal images of cell surface GABA_B_R2 labelling in neurons co-transfected with eGFP, GABA_B_R1 and wild-type (WT) or variant GABA_B_R2 (*inset*) to drive over-expression. (**B**) Normalized (to R1R2 wild-type = 100%, left ordinate) and raw (right ordinate) cell surface fluorescence intensities of wild-type or variant R1R2 or cells expressing GFP-only from **A**. (**C**) Representative K^+^ currents in response to 10 and 100 μM baclofen recorded from hippocampal neurons expressing eGFP with or without wild-type or variant R2 (pictogram) or untransfected cells. (**D**) Mean baclofen-activated K^+^ current density of neurons expressing wild-type or mutant GABA_B_R2. *P < 0.05, **P < 0.01, ***P < 0.001, one-way ANOVA with Tukey–Kramer test or non-parametric ANOVA with Dunn's multiple comparison test.

GABA_B_Rs execute their physiological roles from the neuronal plasma membrane, and thus the expression levels of each variant were also assessed in the native environment of hippocampal neurons by transiently co-expressing GABA_B_R2^flag^ with eGFP in cultures. To efficiently express at the cell surface, exogenous GABA_B_R2^flag^ subunits must heterodimerize with endogenous GABA_B_R1, thereby limiting their overexpression. Using this strategy, even though cell surface staining of wild-type receptors was detected (Supplementary Fig. 2A and B), no plasma membrane expression was resolved for the mutants in neurons [*F*(4,176) = 86.6, *P* < 0.001; KW one-way ANOVA]. To aid heterodimeric co-assembly^48^ while further investigating the absence of neuronal cell surface expression of the R2 variants, GABA_B_R1a and GABA_B_R2^flag^ were co-expressed to facilitate cell surface expression by driving overexpression. However, under these conditions, R2 variant expression was still severely compromised compared to wild-type receptors [*F*(4,217) = 111.35, *P* < 0.001, KW one-way ANOVA; Fig. 4A and B]. Cell surface expression levels of overexpressed S695I and I705N remained unchanged compared to eGFP-only expressing neurons (*P* > 0.05), while expression of overexpressed G693W was only marginally increased (*P* < 0.05) compared to eGFP-expressing cells.

These results show that while R2 variant plasma membrane expression is largely intact in heterologous HEK-293 T cells, a contrasting severe reduction of neuronal cell surface expression typifies these GABA_B_R disease variants. Thus, the R2 variants prevent GABA_B_R expression, which may result in a marked reduction of GABAergic signalling.

### Variants reduce GABA_B_R signalling in hippocampal neurons

The functional consequences of reduced neuronal cell membrane GABA_B_R expression were assessed in hippocampal neurons expressing wild-type or variant GABA_B_R2 along with eGFP. We activated GIRK currents in neurons using the specific GABA_B_R agonist baclofen.

Sequentially expressing just the GABA_B_R2 variants in the absence of exogenous GABA_B_R revealed substantive changes to inwardly-rectifying K^+^ currents. The current density for S695I-expressing neurons was reduced compared to untransfeced cells (*P* < 0.05), eGFP only controls (*P* < 0.001) and neurons expressing wild-type R2 (*P* < 0.001) in response to 10 μM baclofen [*F*(5,154) = 31.23, *P* < 0.0001, KW one-way ANOVA]. At maximal baclofen concentrations (100 μM), the current density of S695I was again lower compared to eGFP controls or wild-type R2 expressing neurons [*F*(5,158) = 28.6, *P* < 0.0001, KW one-way ANOVA]. This indicated that expressing S695I may have a negative effect on the function of endogenous and wild-type GABA_B_Rs.

These results were corroborated by co-expressing GABA_B_R2 and GABA_B_R1 (Supplementary Fig. 2C and D). The current densities associated with G693W and I705N were unaffected for neurons expressing R1 and R2 compared to wild-type receptors (*P* > 0.05), suggesting that the mechanism by which G693W and I705N increase neural excitability does not involve depressing wild-type R2 expression (Supplementary Fig. 2C and D*)*. However, current density for S695I was lower compared to untransfected neurons at 10 (*P* < 0.05) and 100 μM (*P* < 0.001) baclofen as well as eGFP-only controls (*P* < 0.001) and wild-type R2-expressing neurons (*P* < 0.001) at 100 μM baclofen [10 μM *F*(5,122) = 57.07; 100 μM *F*(5,122) = 55.5; *P* < 0.0001, KW one-way ANOVA; Supplementary Fig. 2C and D] consistent with a negative role of S695I on endogenous wild-type subunit expression.

By examining the functional properties of the R2 variants, especially S695I and I705N, it is likely that the profound effect on GABAergic signalling occurs via a consequent reduction of GABA_B_R function, and that S695I seemingly acts to suppress wild-type GABA_B_Rs to further reduce receptor signalling.

### Postsynaptic excitatory neurotransmission unchanged by S695I

We focused this part of our study on S695I because of its effectiveness in reducing GABA_B_R activity evident in a heterologous expression system designed to avoid overexpression of the R2 variant.

To investigate the impact of S695I on excitatory neurotransmission in hippocampal cultured neurons, we used whole-cell recording with a Cs-methanesulfonate-based patch electrode solution to block postsynaptic GABA_B_R-activated GIRK channels^49,50^ in the presence of picrotoxin and tetrodotoxin. For neurons expressing just R2 S695I, the mEPSC frequency [*F*(2,22) = 0.997, *P* = 0.3853], amplitude [*F*(2,27) = 2.9 KW, *P* = 0.2301] and kinetics [rise time: *F*(2,27) = 4.8 KW, *P* = 0.0891; T50: *F*(2,27) = 2.8, *P* = 0.0767; τ: *F*(2,27) = 3.3 KW, *P* = 0.1897; area: *F*(2,27) = 2.2, *P* = 0.1345] all remained unchanged compared to wild-type R2 expressing neurons (one-way ANOVA; Supplementary Fig. 3).

**Figure 5:**
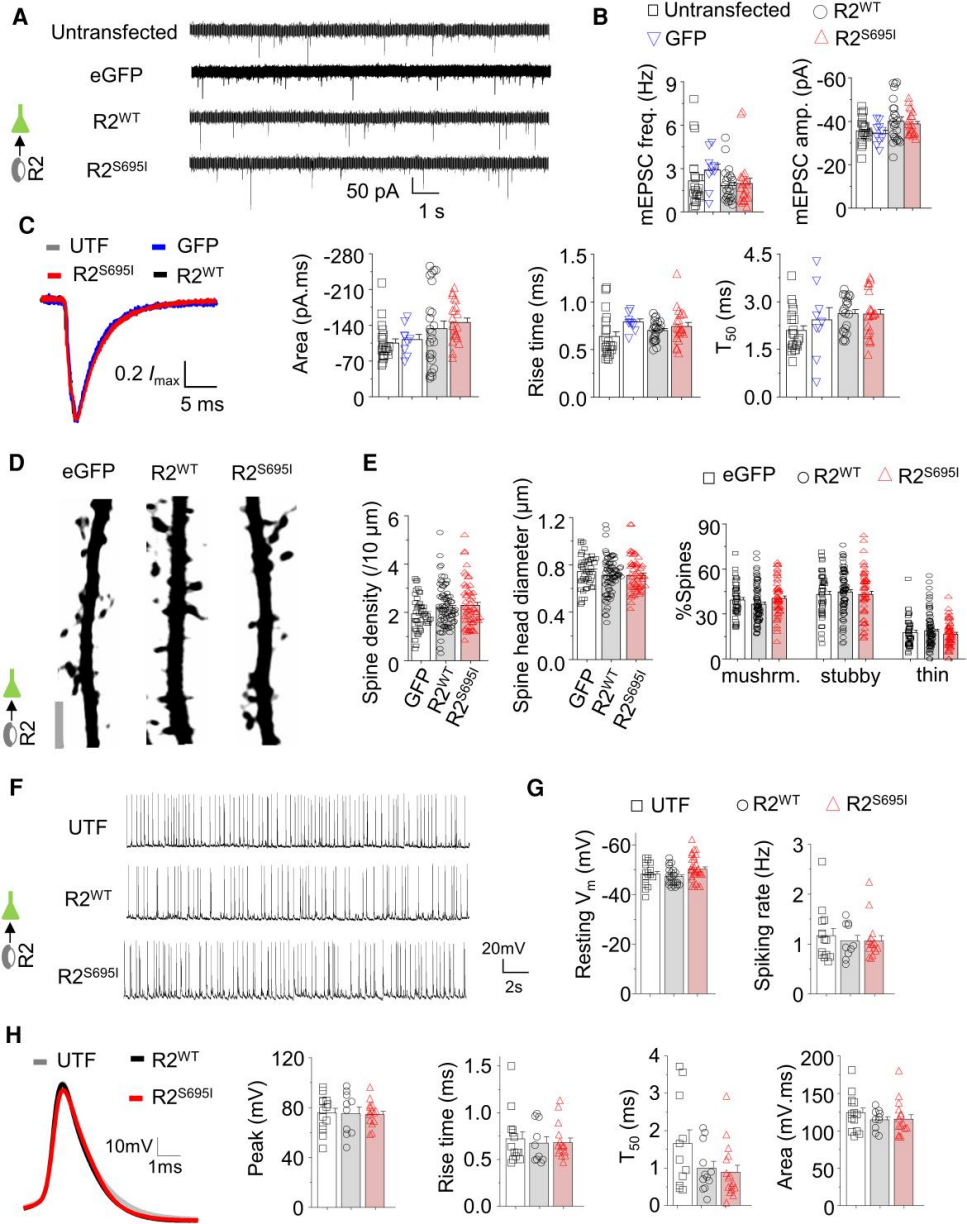
**Excitatory neurotransmission and GABA_B_R2 variants.** (**A**) Representative miniature excitatory postsynaptic currents (mEPSCs) recorded from hippocampal neurons that are untransfected or expressing eGFP alone or with wild-type or S695I GABA_B_R2 (*inset*). (**B**) Average frequency and amplitude of mEPSCs for cells treated as in **A**. (**C**) Average mEPSC waveforms and mean charge transfer, rise time and T_50_ of kinetics for mEPSCs recorded from neurons in **A**. (**D**) Representative images of neuronal dendrites expressing eGFP alone or in combination with wild-type or S695I (pictogram) showing spines. (**E**) Density, head size and proportion of mushroom (mushrm.), stubby and thin spines in hippocampal neurons treated as shown in **D**. (**F**) Representative spontaneous action potentials recorded by whole-cell current clamp from untransfected (UTF) and wild-type- or S695I- expressing GABA_B_R2. (**G**) Average resting membrane potential and spiking rate of hippocampal neurons. (**H**) Average action potential waveforms, peak, rise time, T_50_ and charge transfer of spikes. *P < 0.05, one-way ANOVA with Tukey test or non-parametric Kruskal–Wallis ANOVA with Dunn's multiple comparison test. Scale bar = 5 μm.

Reverting to a K^+^-based internal solution (K-gluconate) to preserve GIRK channel function allowed the postsynaptic modulation of mEPSCs by GABABRs to be probed. Under these conditions, mEPSC frequency [*F*(3,71) = 6.36 KW, *P* = 0.0953] and amplitude [*F*(3,71) = 2.32, *P* = 0.0825], as well as kinetics [charge transfer: *F*(3,71) = 2.41, *P* = 0.0737; rise time: *F*(3,71) = 2.2, *P* = 0.0901; T50: *F*(3,71) = 2.524, *P* = 0.0646], remained unchanged (one-way ANOVA; Fig. 5A–C), suggesting overall that the expression of the GABA_B_R2 variant in postsynaptic cells had no clear effect on glutamatergic neurotransmission.

Any subtle changes to glutamatergic neurotransmission may be associated with altered dendritic spine structure since these form the sites of glutamatergic inputs on dendrites.^51^ Imaging hippocampal neurons expressing either wild-type R2 or S695I, with eGFP, revealed unchanged spine density [*F*(2,179) = 3.434 KW, *P* = 0.1796], spine diameter [*F*(2,179) = 1.94 KW, *P* = 0.4565], or the proportions of mushroom [*F*(2,180) = 5.063 KW, *P* = 0.0795], stubby [*F*(2,180) = 0.2886, *P* = 0.7496] or thin [*F*(2,180) = 0.4149 KW, *P* = 0.8126] spines (Fig. 5D and E).

To explore any physiological consequences of expressing S695I, we probed action potential firing in hippocampal neurons expressing either wild-type R2 or S695I. Spontaneous firing frequency [*F*(2,36) = 2.69, *P* = 0.0813] and the peak amplitudes of action potentials [*F*(2,36) = 0.03, *P* = 0.9664, one-way ANOVA] remained unchanged; the resting membrane potential [*F*(2,70) = 4.923 KW, *P* = 0.0853; Fig. 5F–H] was similarly unchanged between untransfected, wild-type R2- or S695I-expressing neurons. Moreover, unitary spike area [*F*(2,36) = 0.7175; *P* = 0.4948], rise time [*F*(2,36) = 0.4257 KW, *P* = 0.8083] and the action potential repolarization phase, exemplified by T_50_ [*F*(2,36) = 0.1929 KW; *P* = 0.9081] between wild-type R2 and S695I (Fig. 5H) was also unchanged.

Combined, these findings suggest that S695I affects neither dendritic spine structure nor glutamatergic EPSCs and intrinsic membrane properties, as described previously for GABA_B_Rs under basal conditions.^52-55^ Thus, the expression of S695I has had no decisive effects on postsynaptic glutamatergic neurotransmission and spike firing in cultured neurons.

### Elevated presynaptic Ca^2+^ signalling due to S695I

Since an equally important role for GABA_B_Rs is to control neurotransmitter release at presynaptic terminals, the impact of S695I on presynaptic Ca^2+^ transients was addressed using the Ca^2+^ reporter, GCaMP6*f* fused to synaptophysin (synaptophysin-GCaMP6*f*).^25^

**Figure 6:**
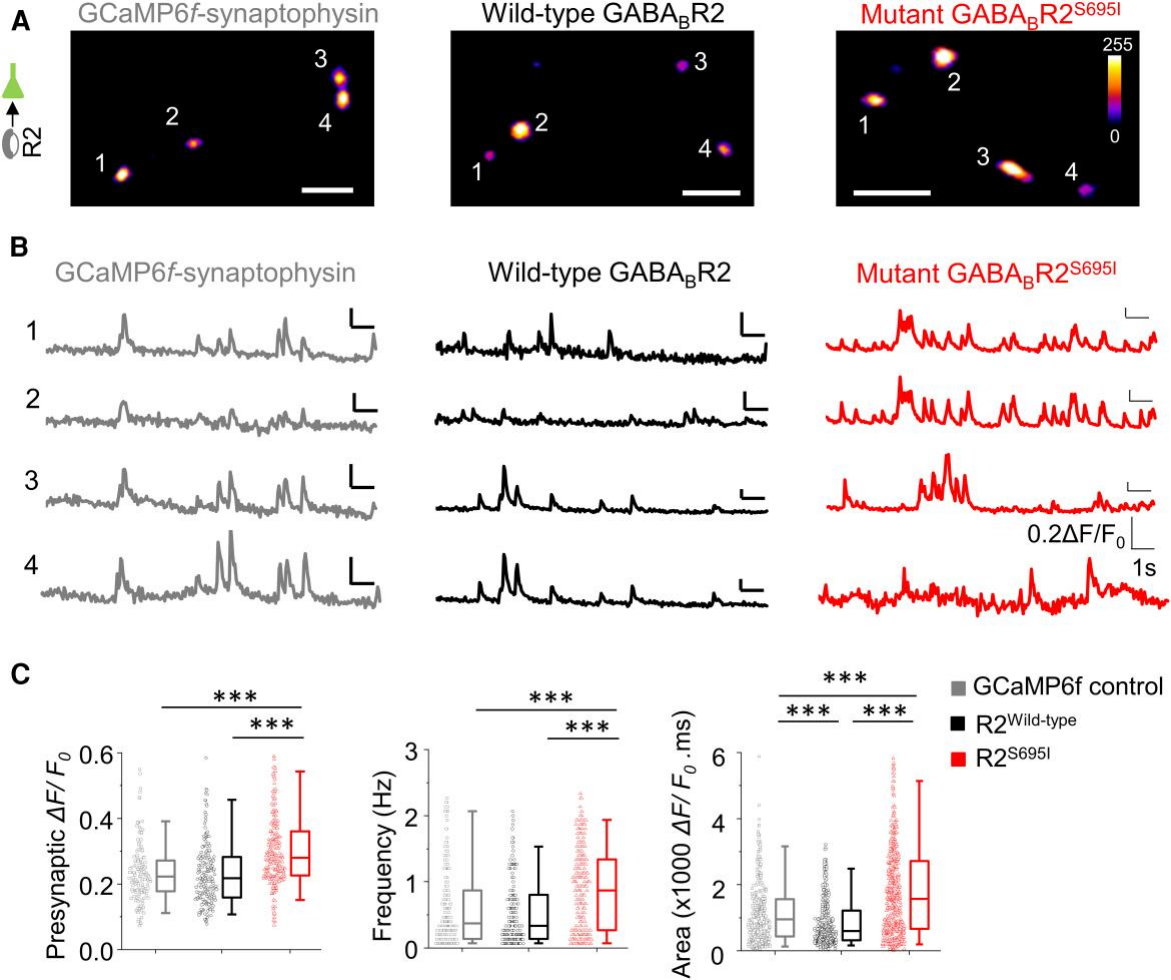
**Elevated presynaptic Ca^2+^ transients due to GABA_B_R variants.** (**A**) Representative images of presynaptic Ca^2+^ signals in neurons expressing synaptophysin-GCaMP6*f* alone (*left*) or with wild-type (*middle*) or S695I GABA_B_R2 (*right*). (**B**) Example recordings of Ca^2+^ transients from presynaptic terminals in **A**. (**C**) Median presynaptic ΔF/F_0_, frequency of Ca^2+^ transients and area under the curve for fluorescence change in a 15 s epoch per presynaptic terminal in wild-type and S695I GABA_B_R2 expressing nerve endings. ***P < 0.001, non-parametric Kruskal–Wallis ANOVA with Dunn's multiple comparisons test. Scale bars = 5 μm.

Expression of synaptophysin-GCaMP6*f* allowed spontaneous Ca^2+^ transients to be imaged in presynaptic terminals (Supplementary Video 1). Co-expression with wild-type GABA_B_R2 neither altered (*P* > 0.05) the mean ΔF/F_0_ [*F*(2,569) = 63.4, *P* < 0.001, KW one-way ANOVA] nor the frequency [*F*(2,569) = 36.6, *P* < 0.001, KW one-way ANOVA; Fig. 6A–C] of presynaptic Ca^2+^ transients compared to synaptophysin-GCaMP6*f* only expressing neurons. However, the area under the ΔF/F_0_ curve of Ca^2+^ activity during the imaging epoch was reduced due to the expression of wild-type R2 compared to synaptophysin-GCaMP6*f* alone [*F*(2,1280) = 147.2, *P* < 0.001, KW one-way ANOVA; Fig. 6A–C]. Although this appears to contrast with the findings from our whole-cell EPSC and spike firing recordings, pre- and postsynaptic signalling could be differentially sensitive to exogenous expression-related changes in GABA_B_R expression levels.

By contrast to wild-type R2, expression of R2-S695I increased the ΔF/F_0_ (*P* < 0.001), the frequency of Ca^2+^ transients (*P* < 0.001), and the area under the curve compared to R2-wild-type or synaptophysin-GCaMP6*f* alone controls (*P* < 0.001, one-way ANOVA) suggesting that increased presynaptic Ca^2+^ activity, due to S695I expression, could be a key mechanism by which this variant orchestrates its pathophysiological phenotype.

### Reversal of presynaptic GABA_B_R signalling defects by positive allosteric modulation

**Figure 7:**
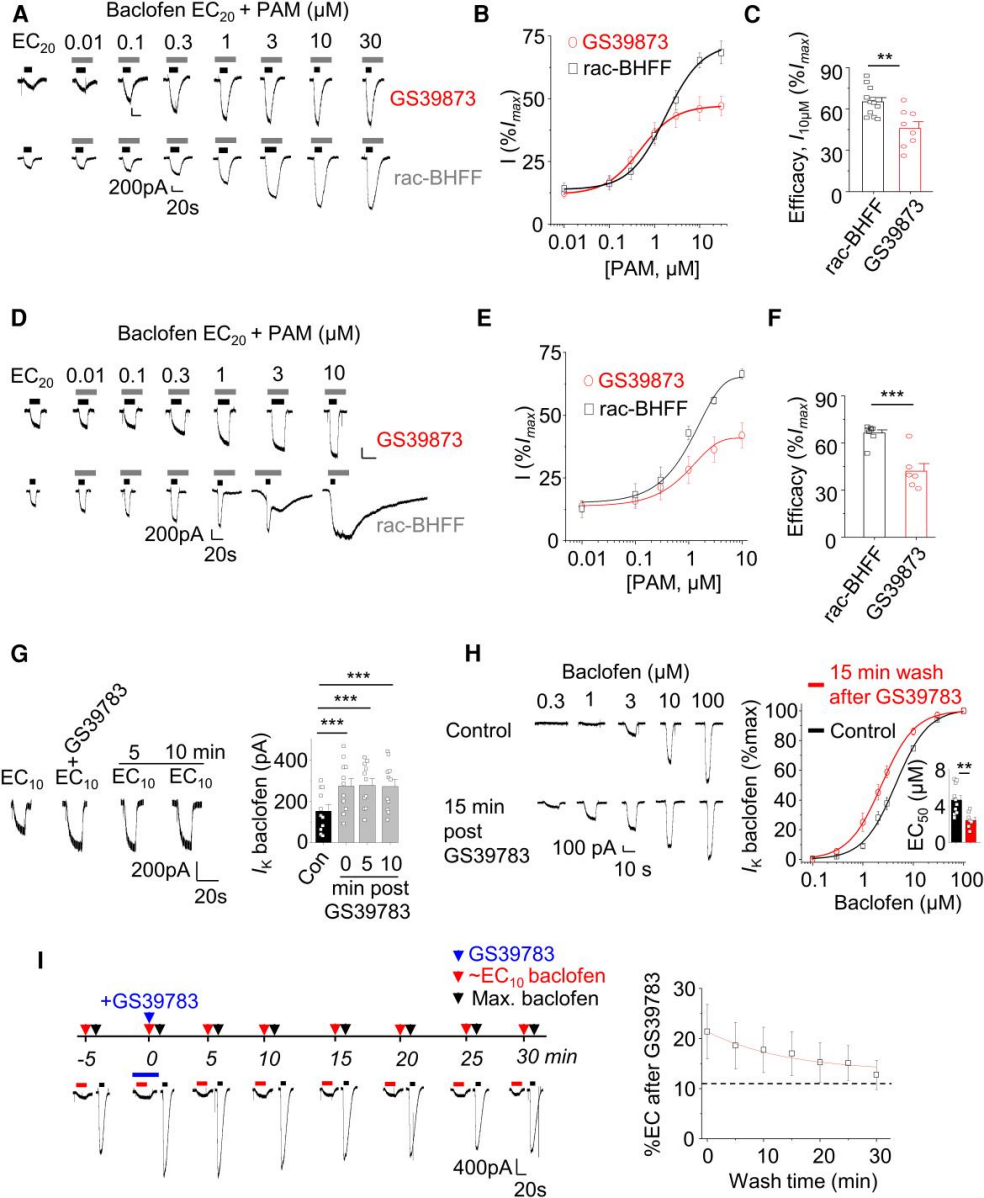
**Pharmacological characterization of GABA_B_R positive allosteric modulators (PAMs).** (**A**) Whole-cell GIRK currents activated by ∼EC_20_ baclofen showing potentiation by GS39783 and rac-BHFF in GABA_B_R1R2-transfected GIRK cells. (**B**) PAM potentiation curves for GS39783 and rac-BHFF normalized (%) to the maximal baclofen responses from the same cell (=100%). (**C**) Bar graph comparing maximal potentiation by GS39783 (30 μM) and rac-BHFF (30 μM). (**D**) Whole-cell GIRK currents activated by ∼EC_20_ baclofen showing potentiation by GS39783 and rac-BHFF in hippocampal neurons in culture at 14–21 days *in vitro*. (**E**) Potentiation curves for GS39783 and rac-BHFF normalized to maximal baclofen responses (=100%) from the same neuron. (**F**) Bar graph comparing maximal potentiation of GS39783 and rac-BHFF. (**G**) Example recordings of ∼EC_10_ baclofen prior to, during and 5 or 10 min after 3 μM GS39783 application in hippocampal neurons. Bar chart showing increased ∼EC_10_ 10 min after the cessation of GS39783 application. (**H**) Example recordings of baclofen-activated currents in hippocampal neurons before and 15 min after application of 3 μM GS39783. Note the left-shifted concentration response curve and lower EC_50_ after 15 min wash (*inset*). (**I**) Protocol and example recordings showing ∼EC_10_ and maximum baclofen currents measured before, during and up to 30 min after the application of 3 μM GS39783 along with a %EC_10_ decay curve (*right*) for baclofen after GS39783 application. Dotted line shows the initial ∼EC_10_ value prior to GS39783 application. *n* = 6–12. **P < 0.01, ***P < 0.001, two-tailed unpaired *t*-test, Mann–Whitney test or repeated measures ANOVA with Tukey–Kramer multiple comparisons test (**G**).

We reasoned that increasing GABA_B_R activity could compensate for the deleterious effects of S695I and thus recover a ‘ground state’ for presynaptic receptor signalling. To increase GABA_B_R signalling, we examined the PAMs, GS39783 and rac-BHFF for their effectiveness in GIRK cells (Fig. 7A–C) and hippocampal neurons (Fig. 7D–F) by constructing PAM potentiation curves for the response to ∼EC_20_ baclofen. Rac-BHFF was consistently more efficacious than GS39783 in GIRK cells (*P* = 0.0021, two-tailed unpaired *t*-test) and hippocampal neurons (*P* = 0.0013).

Interestingly, in hippocampal neurons, GS39783 was slow to wash off (Fig. 7G and H) with potentiated baclofen responses, following a single 3 μM submaximal GS39783 exposure, reduced by only ∼5% after 10 min of washing [*F*(3,30) = 9.9, *P* = 0.0001, repeated measures ANOVA; Fig. 7G]. The baclofen EC_50_ post-GS39783 (EC_50_ = 2.43 ± 0.3 µM) remained lower even after a 15 min wash compared to the control pre-GS39783 EC_50_ (4.7 ± 0.4 µM; *P* = 0.0029, *n* = 7–11; Fig. 7H). Intriguingly, the recovery kinetics (τ = 14.2 ± 9.6 min; Fig. 7I) for GS39783 potentiation during the wash-off phase, studied in GIRK cells by applying consecutive pairs of ∼EC_10_ and maximal baclofen concentrations every 5 min following a single exposure to 3 μM GS39783, is similar to the rate of internalization for cell surface GABA_B_Rs (τ = 13.4 ± 1.4 min)^12^ in the same cells. This similarity may imply that GS39783 binds tightly to the GABA_B_R and that potentiation is only terminated predominantly via endocytosis instead of PAM unbinding.

**Figure 8:**
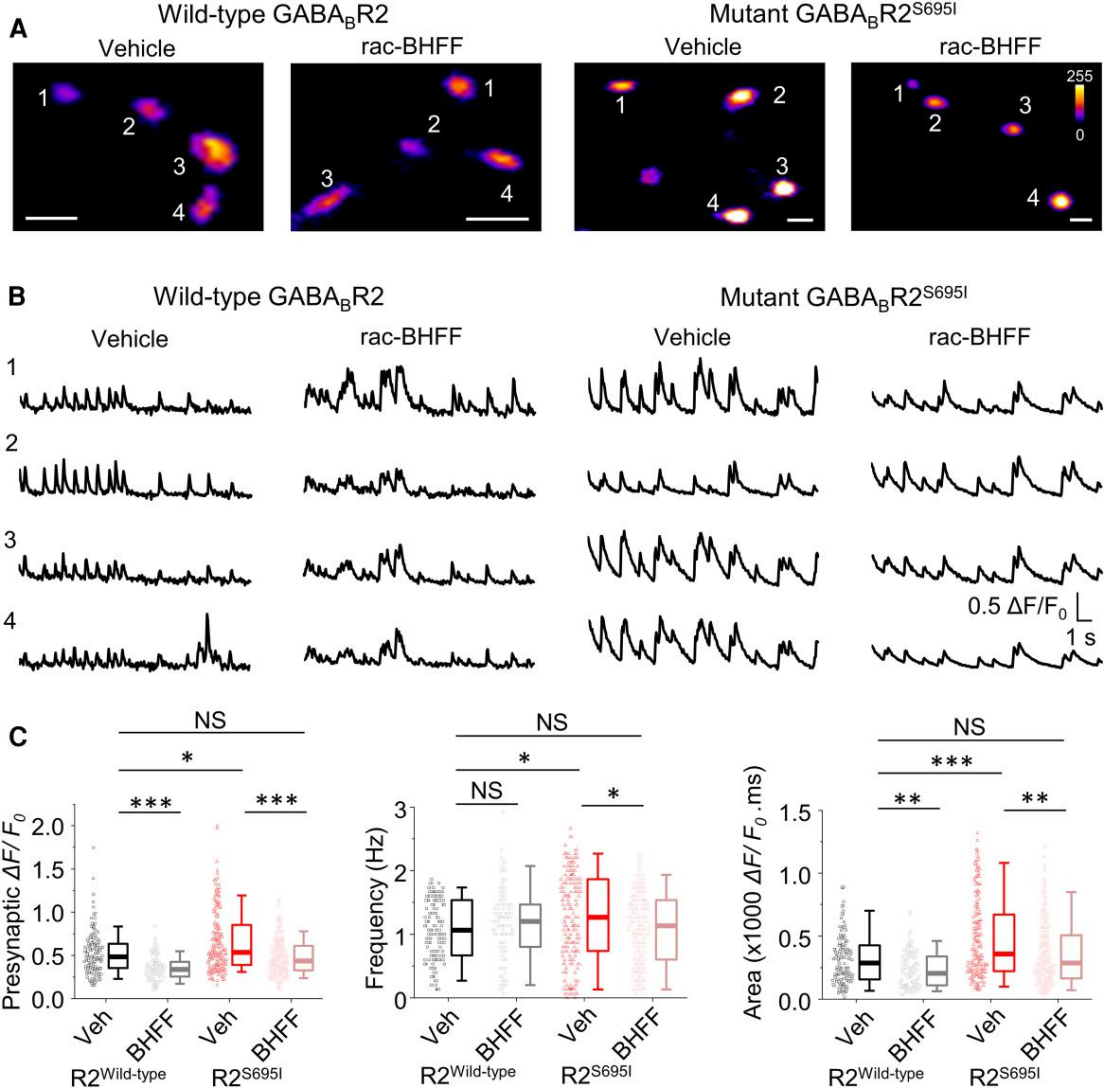
**Reversal of presynaptic increased activity with a GABA_B_R positive allosteric modulator (PAM).** (**A**) Images of presynaptic Ca^2+^ signals in neurons expressing synaptophysin-GCaMP6*f* with wild-type or S695I GABA_B_R2 in the presence of a vehicle control or 1 μM rac-BHFF. For fluorescing termini, the intensity (ΔF/F_0_) of the Ca^2+^ transients was reduced by rac-BHFF for both termini expressing wild-type or variant R2. (**B**) Example Ca^2+^ transient recordings from the presynaptic terminals in **A**. (**C**) Median presynaptic ΔF/F_0_, Ca^2+^ transient frequency and area for the Ca^2+^ signals recorded in 15 s epochs per presynaptic terminal in wild-type and S695I GABA_B_R2 expressing nerve endings in vehicle or rac-BHFF. *P < 0.05, **P < 0.01, ***P < 0.001, NS = not significant, non-parametric Kruskal–Wallis ANOVA with Dunn's multiple comparisons test. Scale bars = 2 μm.

Owing to the pseudo-irreversible nature of GS39783, we therefore used rac-BHFF at a low concentration (1 μM) to negate the deleterious functional effects of S695I. Applying rac-BHFF reduced both wild-type (*P* < 0.001) and S695I (*P* < 0.001) ΔF/F_0_ [*F*(3,775) = 89.7 KW, *P* = 0.007, one-way ANOVA; Fig. 8A–C and Supplementary Video 2]. Despite our homology modelling proposing that BHFF binding could be affected in the R2-S695I mutant, this PAM normalized the difference in Ca^2+^ transients between wild-type and rac-BHFF-treated S695I axon termini (*P* > 0.05). The frequency of presynaptic transients in rac-BHFF was unaltered for wild-type neurons (*P* > 0.05) but reduced (*P* < 0.05) in S695I-expressing neurons effectively normalizing (*P* > 0.05) the frequency to wild-type levels [*F*(3,778) = 12.2, *P* = 0.007, KW one-way ANOVA]. Finally, the overall area under the curve was also reduced by rac-BHFF for wild-type (*P* < 0.01) and S695I (*P* < 0.01) with no difference (*P* > 0.05) between wild-type compared to rac-BHFF treated S695I termini [*F*(3,732) = 59.4, *P* < 0.001, KW one-way ANOVA].

Together, these results confirm that elevated presynaptic Ca^2+^ signalling due to reduced GABA_B_R function caused by S695I can be rescued *in vitro* by positive allosteric modulation. This may have important implications for treating the phenotypes associated with neurodevelopmental disorders.

## Discussion

GABA receptor genetic variants are increasingly linked to neurodevelopmental disorders such as epileptic encephalopathy, autism spectrum disorders, intellectual disability, global developmental delay and Rett syndrome^56-58^, which are often comorbid. Aside from the three variants studied here, other pathogenic GABA_B_R2 variants can cause variable intellectual disability with or without seizures or Rett-like phenotypes. Interestingly, these variants are also located within the receptor's transmembrane^13,14,16-18,20-24,59,60^ or N-terminal domains,^14,15,19^ emphasizing their importance in these disorders. Cryo-EM structures of GABA_B_Rs reveal detailed insights into their conformational states.^32-35^ From such structures, TM6 is deemed crucial for receptor activation and forms an integral part of GS39783 and rac-BHFF PAM binding pockets resembling the evolutionary conserved agonist-(orthosteric) binding site in class-A GPCRs.^61^ When expressed on the cell surface, N-terminal variants (R212Q, T394 M, G440R) are likely to affect GABA binding and the activation of signal transduction, while variants located within TM4 (A567T), TM5 (M668L) and TM6 (G693W, S695I, I705N, A707T) will affect signal transduction and G-protein coupling. Therefore, a diverse set of mechanisms will underlie variant phenotypes in neurodevelopmental disorders, and clinical manifestations will depend on additional contributions from genetic composition, penetrance and variability.

### Perturbing GABA_B_R signalling and trafficking with R2 variants

GABA_B_R subunit variants have previously been characterized in heterologous expression systems.^20,22^ Here, we used whole-cell electrophysiology and flow cytometry as first-order approaches to interrogate the pharmacological and trafficking properties of the GABA_B_R2 variants. The reduced maximal GABA currents for G693W, S695I and I705N in GIRK cells are consistent with impaired signalling.^20^ Lower currents for I705N in these cells are partly due to reduced cell surface expression of GABA_B_R2 variant receptors, irrespective of whether the R2 homomer or R1aR2 heterodimer is studied. Using ELISA^22^ or immunofluorescence^20^ assays, prior reports concluded that I705N expression was unaffected; however, the resolution of the flow cytometry method used here detected a reduction. The minimal GABA sensitivity of GABA_B_R2 S695I and reduced G693W maximal currents in GIRK cells in the absence of cell surface expression changes indicate signal transduction defects. Biochemical evidence from immunoprecipitation did not reveal changes to heterodimerization for S695I, and therefore this mutant likely suffers from defective transduction of ligand-binding signals. Expression of wild-type R1 with R2-S695I in *Xenopus* oocytes, which permit expression of a wide variety of difficult-to-translate constructs,^30^ resolved GABA-activated GIRK currents, albeit with reduced maximal currents and altered kinetics compared to wild-type receptors.^46^ Therefore, these GABA_B_R2-variants are characterized by transduction defects that reduce GABA_B_R signalling.

Counter-intuitively, the low efficacy R2-variant GABA_B_Rs also exhibit greater sensitivity to GABA (lower EC_50_s). Structural modelling tentatively indicates that this may be due to repositioned R2-variant TMDs, which for S695I could involve stabilization of active structures that may underlie changes to GABA sensitivity.^62^ Using chimeric Gα_qi_ assays, higher constitutive activity has been reported for S695I and I705N,^22,62^ but others have not reproduced these results.^61^ Such a mechanism is unlikely to offer protection against seizures as these receptors also show severely impaired cell surface expression in neurons.

By studying GABA_B_R2 variants in neurons, for the first time, to our knowledge, we propose that trafficking defects in GABA_B_R2 is another principal mechanism by which these receptors could cause dysfunction. Our results provide a plausible molecular-level explanation by which GABA_B_R2 variants could orchestrate seizures and neurodevelopmental deficits due to reduced neuronal GABA_B_R signalling. Similarly, knocking out GABA_B_R2,^63^ or treatment with GABA_B_R antagonists^64,65^ or by perturbing GABA_B_R cell surface delivery^66^ all result in seizures, highlighting the importance of maintaining appropriate levels of functional GABA_B_Rs at the cell surface.

### Cell surface expression of R2 variants

A key unexpected finding from our study was the difference in cell surface expression for the R2 variant receptors when expressed in neurons compared to heterologous cells. Neurons are known for their stringent quality control of cell surface expression.^30^ Moreover, the role of TM6 is clearly important as single amino acid substitutions prevent cell surface trafficking of variant receptors in neurons but not in HEK-293 cells. Our findings suggest that TM6 could form a critical part of a neuronal quality control checkpoint that determines plasma membrane expression of GABA_B_Rs. Structural modelling of each GABA_B_R2 variant predicted subtle changes to side-chain rotamers for the variant residues that presumably are disruptive to TM6 interactions and thus limiting variant GABA_B_R2 receptor expression at the cell surface. Moreover, subtle changes to the GABA_B_R2 TM6 primary sequence can result in neurodevelopmental disorders. TM6 appears to be a ‘hot spot’ for disease pathogenesis since while G693W, S695I and I705N all cause developmental epileptic encephalopathy, A707 T in the same TMD causes atypical Rett syndrome.^22^ Therefore, characterizing variant receptor properties at synapses in neurons and neural circuits is important for identifying the molecular basis of complex neurodevelopmental disorders.

### Variant R2 and dominant-negative signalling

The first indication of loss-of-function signalling, where expression of a variant subunit also suppressed wild-type receptor function, arose from reduced GIRK current densities in neurons expressing S695I compared to untransfected or eGFP-expressing controls. Driving over-expression of this GABA_B_R2 variant by co-transfection with GABA_B_R1 cDNA caused a greater depression of endogenous currents, confirming the loss-of-function properties of S695I and its effect on the function of wild-type receptors. These effects register the pathological landscape of S695I on the function of native GABA_B_Rs compared to G693W and I705N. The net result for these latter two variants will be reduced GABA_B_R function at pre- and postsynaptic compartments, and for S695I, function will be further reduced due to the inhibitory effect it has on residual wild-type receptor function. In the absence of clinical results and information of patients’ full genetic backgrounds, a direct co-relation of disease severity with GABA_B_R function was unattainable but would be interesting to address.

Mechanistically, S695I could produce its loss-of-function effects by intracellularly sequestering GABA_B_R1 for degradation to reduce the pool of R1 subunits available for heterodimerization with wild-type GABA_B_R2. Our immunoprecipitation studies indicate once co-assembly occurs, this R2 variant may trap heterodimers intracellularly in neurons and accumulated heterodimers containing S695I will be degraded most likely via lysosomal^67^ or proteasomal endoplasmic reticulum-associated degradation.^68^ Formation of higher-order oligomers^69^ and heterodimers with higher stability^62^ should amplify the sequestering impact of S695I manifest by reduced cell surface efficacy of GABA_B_R signalling.

### Functional impact of S695I

GABA_B_Rs control neuronal excitability at pre- and postsynaptic domains, but intrinsic activity, glutamatergic EPSC current amplitude or frequency or structural plasticity of dendritic spines remained unaffected by S695I. However, this accords with previous studies, in which cell spiking^52^ or EPSC amplitudes^53,55^ were unaffected by baclofen. Importantly, the application of GABA_B_R antagonists does not affect EPSC amplitude/ frequency^54^ in hippocampal neurons or action potential spiking,^70^ suggesting overall that postsynaptic GABA_B_Rs are not basally active here. Therefore, a lack of more severe postsynaptic excitability defect due to the expression of S695I is not surprising, as our neurons in culture receive the majority of their inputs from untransfected neurons with wild-type presynaptic signalling profiles. However, the impact of the postsynaptic deficits could be exacerbated in pathological states if multiple inputs show elevated presynaptic signalling and increased glutamate release.

Changes to Ca^2+^ signalling were apparent at presynaptic terminals due to S695I expression. Using the Ca^2+^ sensor GCaMP6*f* localized to presynaptic terminals permitted defects in individual presynaptic boutons to be resolved. Here, as expected, native GABA_B_Rs reduce presynaptic Ca^2+^ transients by inhibiting voltage-gated Ca^2+^ channels.^3^ However, expression of S695I led to increased frequency, amplitude and charge transfer of Ca^2+^ events at presynaptic termini. By comparison, following the expression of wild-type GABA_B_R2, the imaging of single terminals detected a reduction in the presynaptic area of fluorescence transfer without affecting the amplitude or frequency of Ca^2+^ transients.

Taken overall, the mechanism by which GABA_B_R variants generate seizures will likely involve elevated glutamate release from presynaptic terminals. In addition, since elevated GABA release from interneurons can initiate disinhibition and thereby maintain seizures^71^; this could be an additional mechanism by which a lack of GABA_B_R function at interneuron terminals can exacerbate seizures.

### Therapeutic approach for mitigating R2 variant pathogenesis

Treatment of GABA_B_R variant seizures has proven challenging and relies on a combinatorial pharmacological approach. Despite a paucity of treatment options, increasing GABA-mediated signalling is likely to be beneficial. This is evident from indirectly facilitating GABA_B_R signalling for GABA_B_R2 S695I and I705N^20^ with the GABA-transaminase inhibitor, vigabatrin, for a related GABA_B_R2 TM6 variant.^60^ Administering baclofen to activate GABA_B_R signalling is confounded by the incidence of seizures that have been reported in some individuals.^72^ We, therefore, explored an alternative approach using a GABA_B_R PAM to normalize the defects in presynaptic Ca^2+^ signalling for S695I.

Several GABA_B_R PAMs are now known to affect rodent behaviour in preclinical studies of neurological conditions including alcoholism, substance abuse, schizophrenia, anxiety and seizures. PAMs have the advantage of greater temporal specificity compared to agonists as PAMs are (usually) only active in the presence of agonists. Examples of current commercially available PAMs include: CGP7930, GS39783 and rac-BHFF. Rac-BHFF was selected based on its higher efficacy at low concentrations compared to other GABA_B_R PAMs and because CGP7930 is a GABA_A_R PAM and K^+^ channel blocker,^26^ while GS39783 is genotoxic,^73^ in addition to its prolonged binding to GABA_B_Rs. At presynaptic terminals expressing S695I, rac-BHFF normalized the frequency, ΔF/F_0_ amplitude and area of fluorescence transfer for presynaptic Ca^2+^ transients to levels associated with GABA_B_R2 wild-type expressing controls. Rac-BHFF probably achieves this by facilitating the activity of those minimal numbers of wild-type GABA_B_Rs that are expressed on the cell surface in S695I-expressing neurons. We would further predict that GABA_B_R PAMs should have beneficial effects for other variants and neurodevelopmental disorders where receptor expression and signalling is similarly impaired.

Our results highlight the importance of controlling presynaptic excitability in developmental disorders associated with GABA_B_R variants. While increased excitability of interneurons should elevate GABA release, the increased presynaptic release from excitatory neurons will elevate glutamate release, altering neural circuit dynamics to orchestrate seizures.

Overall, we provide proof-of-concept for using PAMs to treat GABA_B_R-associated neurodevelopmental conditions. *In vivo*, rac-BHFF reduces the incidence of audiogenic seizures in mice^74^; thus, testing the effectiveness of PAMs for targeting GABA_B_Rs in neurodevelopmental disorders will be important.

## Supplementary Material

awae232_Supplementary_Data

## Data Availability

The datasets generated and analysed during this current study are included in this published article (and its Supplementary material) and can be made available by the authors on reasonable request.
