# Supplementary material for: Presynaptic hyperexcitability reversed by positive allosteric modulation of a GABABR epilepsy variant
Source: Brain. 2024 Jul 19;148(2):533–48. doi: 10.1093/brain/awae232 (PMC11788220; doi:10.1093/brain/awae232)
Supplement: awae232_Supplementary_Data [file awae232_supplementary_data.zip › brain-2023-02216-File010.pdf]

Supplementary Information

Presynaptic hyperexcitability reversed by allosteric modulation of a $GABA_B$R epilepsy variant

Marielle Minere[1], Martin Mortensen[1], Valentina Dorovykh[1], Gary Warnes[2], Dean Nizetic[2], Trevor G. Smart[1*] and Saad B. Hannan[1,3]

1. Department of Neuroscience, Physiology and Pharmacology, University College London, Gower Street, London, WC1E 6BT, UK

2. Blizard Institute, Barts and The London School of Medicine and Dentistry, 4 Newark Street, London E1 2AT, UK

3. Department of Molecular and Cellular Biology, Harvard University, Cambridge, MA 02138, USA

[*]Correspondence: t.smart@ucl.ac.uk and saadhannan@fas.harvard.edu

Supplementary Figure 1 - Cell surface expression of $GABA_BR$ variants in HEK-293 cells

(A), Representative cytofluorograms of cell surface $GABA_BR1$ expression in HEK-293 cells co-transfected with wild-type or variant $GABA_BR2$. BBS epitope-tagged (green shape) $GABA_BR1$ were labelled with BgTx-Alexa Fluor 555 (red dot in pictogram). ***(B)***, Normalised (left ordinate) and raw (right) cell surface median fluorescence (F; upper panel) and % total cells (normalised and raw; lower panel) in quadrant 2 (Q2) for wild-type and variant receptors. GFP only expressing cells and untransfected cells are also shown. ***(C)***, Cytofluorograms of total expression for BBS-tagged $GABA_BR1$ expressed with wild-type or variant $GABA_BR2$ in HEK-293 cells following membrane permeabilisation (pictogram). ***(D)***, Normalised (left ordinate) and raw (right) median fluorescence (upper panel) and % total cells in Q2 (lower panel) of wild-type and variant $GABA_BR2$ in permeabilised cells. *$P<0.05$, KW one-way ANOVA with Dunn's multiple comparison test.

Supplementary Figure 2 – Impaired cell surface expression and signalling for mutant $GABA_BRs$ in hippocampal neurons

(A), Confocal images of cell surface labelling of flag-tagged wild-type or variant $GABA_BR2$ in hippocampal neurons transfected with cDNAs for R2 and eGFP (pictogram). ***(B)***, Normalised (to R2 wild-type = 100%, left ordinate) and raw (right ordinate) cell surface fluorescence intensities of wild-type or variant $GABA_BR2$ or just GFP-expressing cells from *(A)*. ***(C)***, Representative baclofen-activated K^+ currents from hippocampal neurons expressing wild-type or variant $GABA_BR2$ along with R1 with (pictogram) or without eGFP and untransfected cells. ***(D)***, Mean K^+ current density of neurons expressing R1 and wild-type or variant $GABA_BR2$. *$P<0.05$, **$P<0.01$, ***$P<0.001$, one-way ANOVA with Tukey-Kramer test or nonparametric ANOVA with Dunn's multiple comparison test.

Supplementary Figure 3 – Unchanged excitatory neurotransmission in Cs-based internal solution for S695I-expressing neurons

(A), Representative mEPSCs recorded from hippocampal neurons that are either untransfected (top) or expressing wild-type (middle) or S695I (lower) $GABA_BR2$ along with eGFP (inset). Patch electrodes were filled with a Cs-based internal solution. ***(B)***, Average frequency (left panel) and amplitude (right panel) of mEPSCs for untransfected and R2 expressing neurons. ***(C)***, Average mEPSC peak-scaled waveforms and mean charge transfer, rise time andT_{50} of mEPSCs recorded from neurons. One-way ANOVA with Tukey-Kramer test or nonparametric ANOVA with Dunn's multiple comparison test.

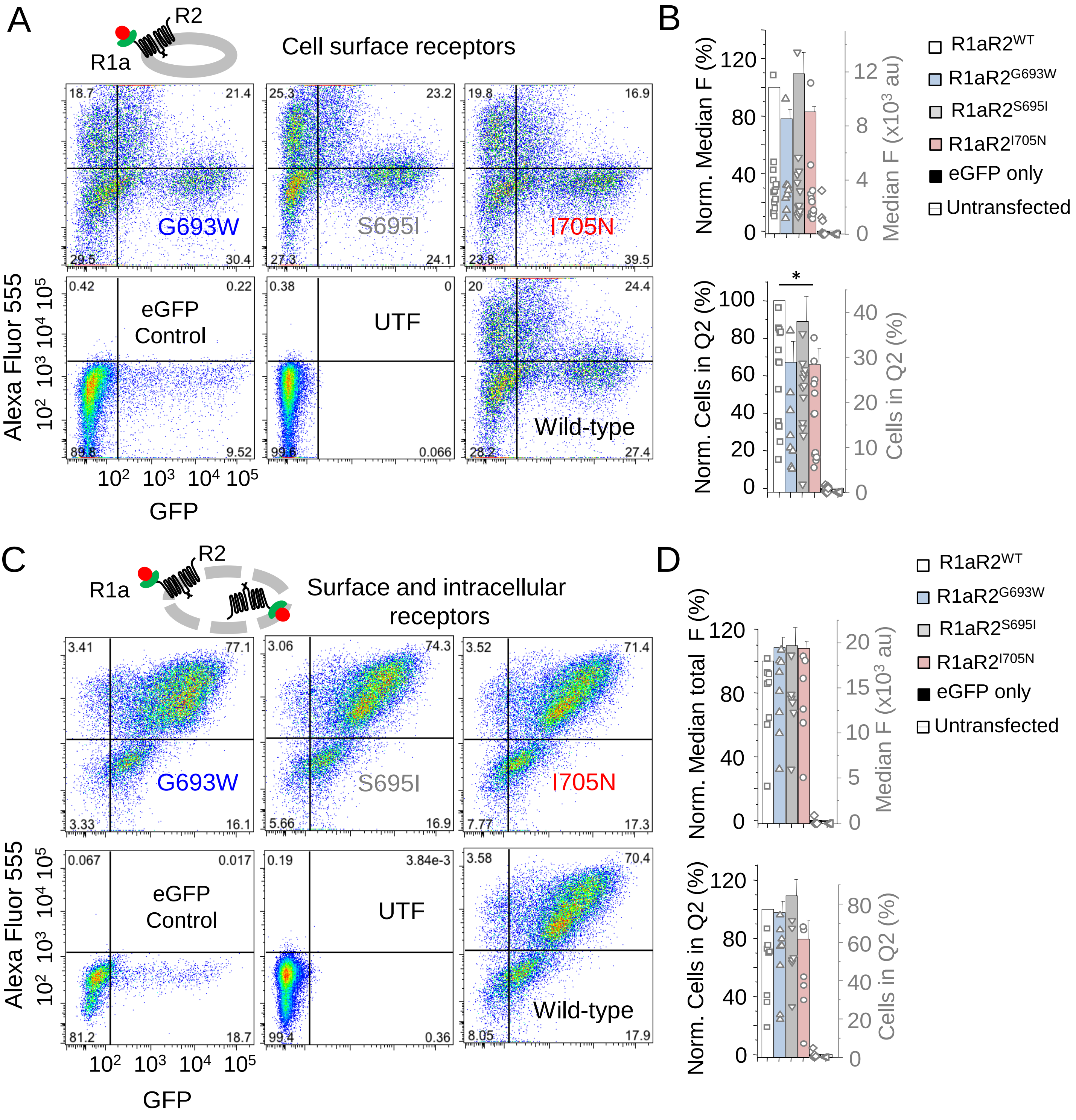

Supplementary Fig 1

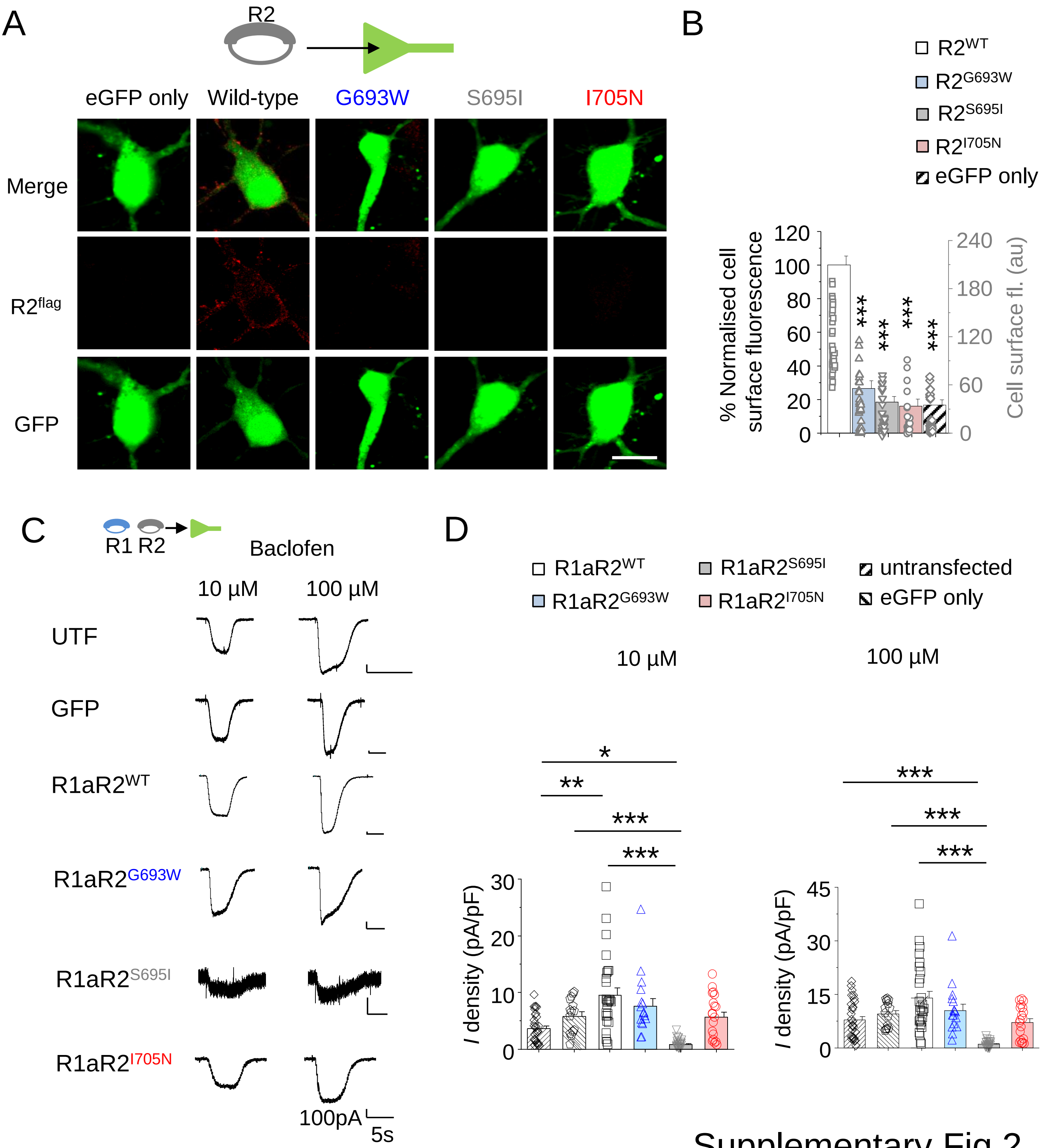

Supplementary Fig 2

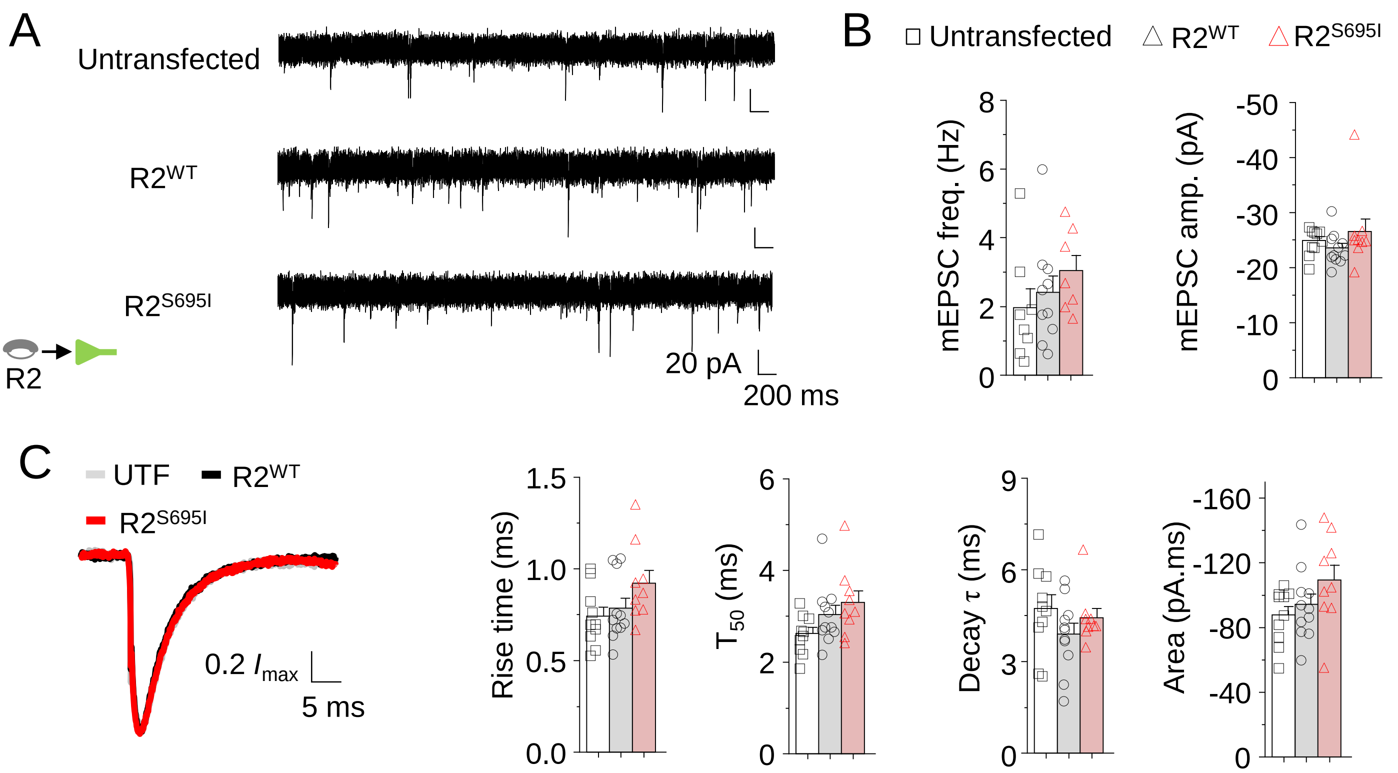

Supplementary Fig 3
