# Supplementary figures and images for: Presynaptic hyperexcitability reversed by positive allosteric modulation of a GABABR epilepsy variant
Source: Brain. 2024 Jul 19;148(2):533–48. doi: 10.1093/brain/awae232 (PMC11788220; doi:10.1093/brain/awae232)

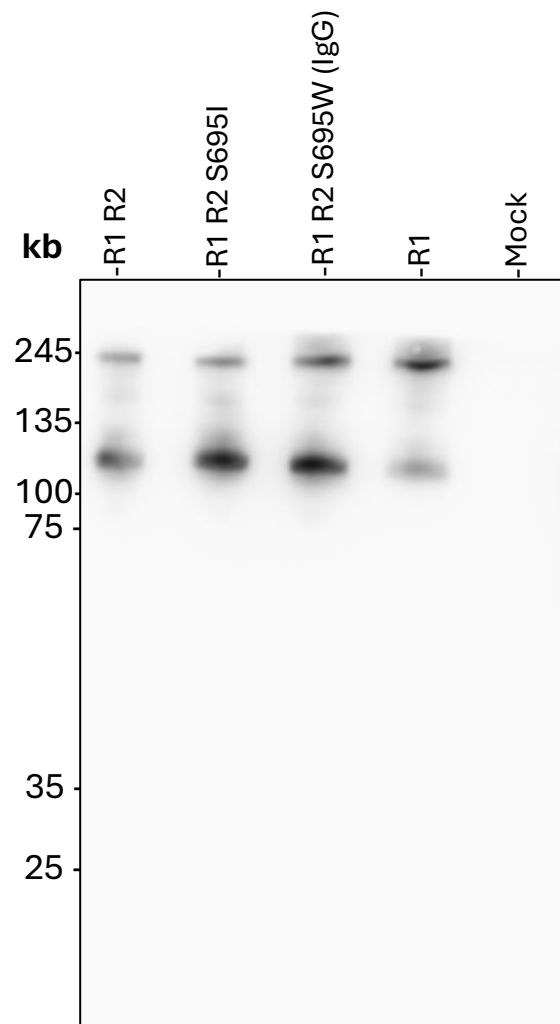

Input  
WB: R1

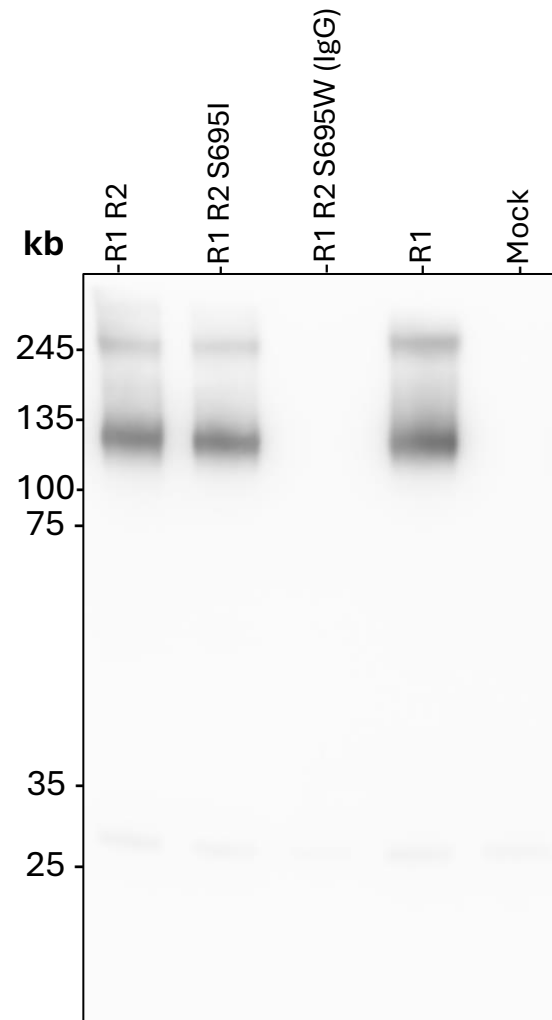

IP: myc  
WB: R1

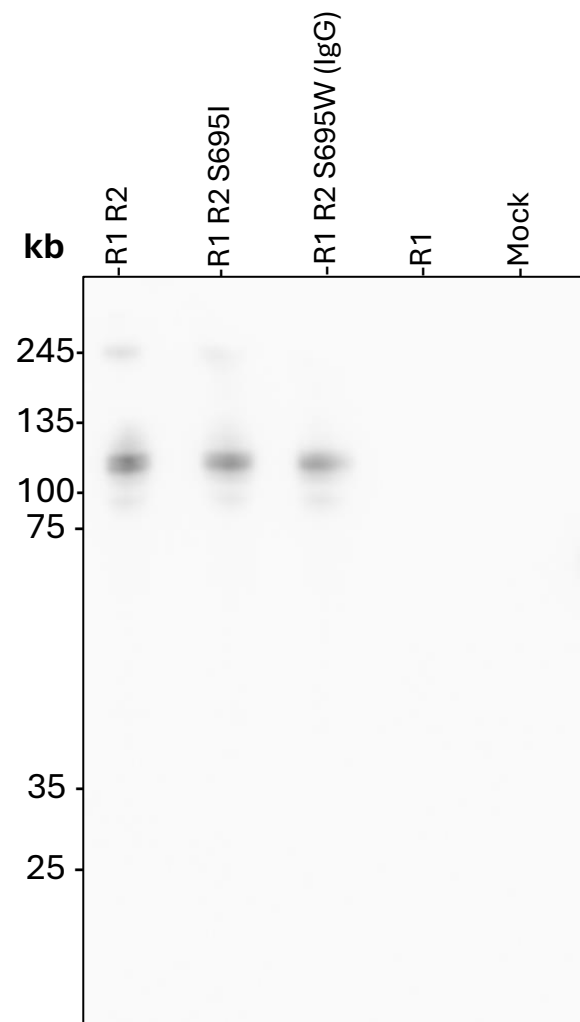

Input  
WB: FLAG

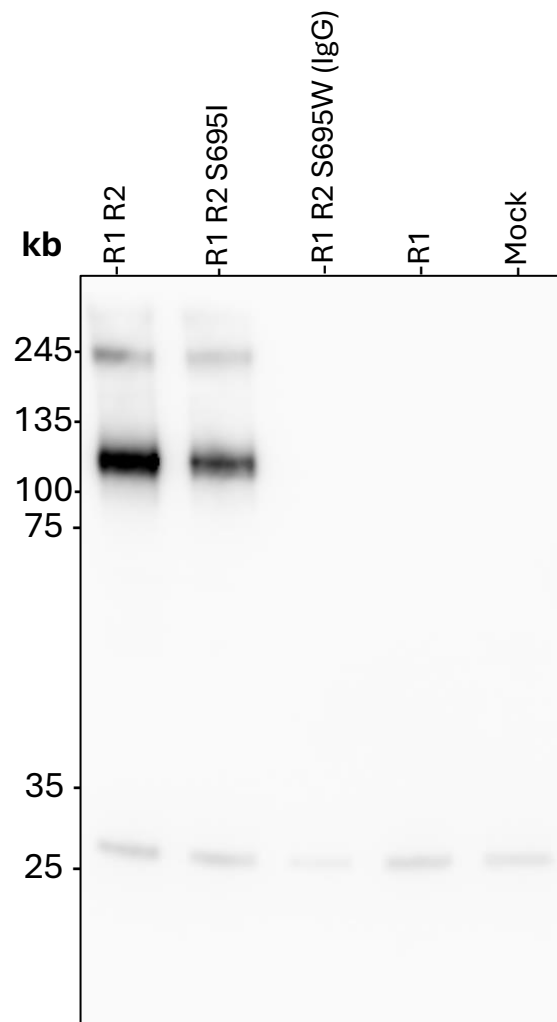

IP  
WB: FLAG

Supplement: awae232_Supplementary_Data [file awae232_supplementary_data.zip › brain-2023-02216-File013.pdf]
